# Diversity in domain architectures of Ser/Thr kinases and their homologues in prokaryotes

**DOI:** 10.1186/1471-2164-6-129

**Published:** 2005-09-19

**Authors:** A Krupa, N Srinivasan

**Affiliations:** 1 Molecular Biophysics Unit, Indian Institute of Science, Bangalore 560 012, India; 2 Cell Cycle Control Laboratory, London Research Institute, Cancer Research – UK, South Mimms, Hertfordshire, EN6 3LD UK

## Abstract

**Background:**

Ser/Thr/Tyr kinases (STYKs) commonly found in eukaryotes have been recently reported in many bacterial species. Recent studies elucidating their cellular functions have established their roles in bacterial growth and development. However functions of a large number of bacterial STYKs still remain elusive. The organisation of domains in a large dataset of bacterial STYKs has been investigated here in order to recognise variety in domain combinations which determine functions of bacterial STYKs.

**Results:**

Using sensitive sequence and profile search methods, domain organisation of over 600 STYKs from 125 prokaryotic genomes have been examined. Kinase catalytic domains of STYKs tethered to a wide range of enzymatic domains such as phosphatases, HSP70, peptidyl prolyl isomerases, pectin esterases and glycoproteases have been identified. Such distinct preferences for domain combinations are not known to be present in either the Histidine kinase or the eukaryotic STYK families. Domain organisation of STYKs specific to certain groups of bacteria has also been noted in the current anlaysis. For example, Hydrophobin like domains in Mycobacterial STYK and penicillin binding domains in few STYKs of Gram-positive organisms and FHA domains in cyanobacterial STYKs. Homologues of characterised substrates of prokaryotic STYKs have also been identified.

**Conclusion:**

The domains and domain architectures of most of the bacterial STYKs identified are very different from the known domain organisation in STYKs of eukaryotes. This observation highlights distinct biological roles of bacterial STYKs compared to eukaryotic STYKs. Bacterial STYKs reveal high diversity in domain organisation. Some of the modular organisations conserved across diverse bacterial species suggests their central role in bacterial physiology. Unique domain architectures of few other groups of STYKs reveal recruitment of functions specific to the species.

## Background

Extracellular signals perceived by the cells invoke an appropriate intracellular response triggering cascades of events that alter the activities of various signalling molecules. The variations in functional states of the proteins are brought about by covalent and non-covalent molecular interactions. Intracellular signalling in prokaryotes and eukaryotes is often achieved by phosphorylation of target proteins. The phosphorylated proteins undergo changes in their three-dimensional (3-D) shapes resulting in altered functional states. Phosphorylation events also lead to changes in the electrostatic interactions and ionisation states of residues at the catalytic site as exemplified by regulation of isocitrate dehydrogenases and HMG-CoA reductase [1,2]. Covalent attachment of the phosphoryl groups to various proteins is catalysed by protein kinases. These enzymes transfer a phosphate group from adenosine tri phosphate (ATP) onto an acceptor amino acid in a substrate protein. The acceptor groups on amino acid residues could be alcoholic as in Ser and Thr, phenolic group as in tyrosine or basic amino acid such as histidine (N^π ^or N^τ^), arginine (guanidino group), and the ε-NH2 group of lysine, carboxyl groups of aspartate and glutamate or thiol group of cysteine [3,4]. In addition to nucleoside triphosphates as donors of the phosphate groups, certain class of phosphorylating enzymes makes use of either phospho-enzyme intermediates or low molecular weight metabolites such as phosphoenol pyruvate, acetyl phosphate or carbamoyl phosphates and polyphosphates as donors [5-7].

Despite the occurrences of various phosphorylating enzymes, cellular signalling mechanisms are dependent on a subset of the list of enzymes mentioned above, for intra-cellular communications. In eubacteria and archaea, two component signal transduction systems [8], also referred to as His-Asp phospho-relay systems, mediate adaptive responses to changes in environmental conditions. The two-component system is composed of a histidine kinase (HK) and a cognate regulatory protein referred to as a response regulator (RR). HKs consist of an ATP-binding kinase domain and the H-box domain carrying the histidine which is the site of phosphorylation. HKs have also been identified in eukaryotes, such as yeast, *Neurospora *and *Arabidopsis thaliana*, and their functional roles have been studied [9,10].

However, in eukaryotes, Ser/Thr and Tyr kinases play a key role in cellular signal transduction. The Ser/Thr and Tyr kinases differ completely from the HKs both in sequence and structure. The Ser/Thr and the Tyr kinase share a common catalytic core of about 270 residues made up of N-terminal lobe consisting of mainly β-strands and a C-terminal lobe composed predominantly of α-helices [11]. The eukaryotes also possess two other distinct classes of protein kinases, the myosin heavy chain kinase/EF-2 kinase [12] and the mitochondrial protein kinases that include the pyruvate dehydrogenase kinase and branched-chain alpha-ketoacid dehydrogenase kinase [13] that differ from the Ser/Thr kinases in sequence and structure. Protein phosphorylation is hence considered to have originated independently in prokaryotes and eukaryotes in order to meet their diverse cellular and environmental requirements.

However studies on the eukaryotic protein-Ser/Thr/Tyr kinase like sequences (STYKs) in the prokaryotes emerged with the identification of proteins phosphorylated on Ser/Thr and Tyr residues in bacteria [14]. Phosphorylation of Ser/Thr residues in archaeal proteins has also been reported for *Sulfolobus acidocaldarius *by earlier studies [15]. Subsequent identification and characterisation of genes encoding STYKs in *Myxococcus xanthus *[16] and *Streptomyces *species [17] confirmed the presence of homologues of eukaryotic protein kinases in bacteria. Following these reports of STYKs, and the release of large number of bacterial genome sequences, a number of groups have surveyed for the occurrences of STYKs in them [18-22]. Their surveys suggested the occurrences of significant number of STYKs in both archaea and eubacterial genomes and they have been further classified based on their sequence similarity to various subfamilies [19]. A previous study [20] proposed that the Ser/Thr/Tyr protein kinases in bacteria have bacterial origin. Kinase activity in the STYKs has been demonstrated in a few species. These studies have suggested the involvement of STYKs in secondary metabolism [17], morphology of the aerial hyphae in *Streptomyces granticolor *[23], regulation of fruiting body formation in *M.xanthus *[24]. STYKs in *Pseudomonas aeruginosa *and *Yersinia pseudotuberculosis *[25,26] are shown to be critical for progression of infection and expression of virulence factors during certain stages of infection. The role of STYKs in the repression of proline utilisation operon and degradation of proline in *S.typhimurium *has been suggested [27].

The recently reported 3-D crystal structures of Mycobacterial PKN-B [28,29] has revealed an overall similarity of their structure to the eukaryotic Ser/Thr protein kinases with variations in the nucleotide binding regions. The prokaryotic homologues also include aminoglycoside kinases which phosphoryate antibiotics, and share remote sequence similarity with the STYKs. The 3-D fold of their catalytic domain is highly similar to the structures of eukaryotic STYKs [30]. Studies on the evolution of STYKs in *Synechocystis *sp. PCC 6803 have proposed the origin of STYKs before the divergence between prokaryotes and eukaryotes during evolution [20]. The conservation of these sequences in significant number of bacterial genomes despite limitation on their genomic size during evolution have therefore suggested their functions are essential in regulation of cellular activities in prokaryotes, as in their eukaryotic counterparts.

The present analysis is focussed on understanding the biological roles of the prokaryotic STYKs which have been shown to be encoded by significant number of genes in a large number of bacterial genomes by various groups. The present study is based on representative domain architectures of STYKs identified from the completely sequenced genomes of 125 prokaryotes which includes 35 genomes compiled in our early version of KinG database [31]. The domain architectures of STYKs and their homologues in 90 additional genomes included in this paper are recently added to the KinG database .

About 600 STYKs and their homologues have been identified in the current study in 125 bacterial genomes, of complete genomic data, using various sequence and profile search methods. Manual analysis of each of these STYKs and homologues has been made in order to check for the presence or absence of residues known to be critical for the activity of eukaryotic protein kinases. Using profile based search method (See *Materials and Methods*) the extent of similarity of bacterial homologues with different subfamilies of eukaryotic protein kinases has been analysed. The analysis suggests that the bacterial homologues form a distinct class of protein kinases as indicated by the highest similarity (20–30% sequence identity) of their catalytic kinase domain with the generic kinase profiles rather than any individual subfamily profile of eukaryotic protein kinases. i.e., the percent sequence identities of bacterial kinases with specific sub-families of eukaryotic STYKs is lower.

During the course of current analysis we encountered lipopolysaccharide kinases (LPSKs) which are well known for their remote relationship with STYKs [32]. RIO1 class of STYKs have been previously identified in eukaryotes [33,34] and in archaea [19] and our earlier work reported the occurrence of RIO1 proteins in all the three major taxons namely, Archaea, Bacteria and Eukaryota [32].

The domain organisation of STYKs identified from various genomes has been studied in an attempt to understand their probable biological roles. Groups of STYKs conserved only among specific classes of bacteria have been identified. Diverse domain arrangements observed in bacterial STYKs are radically different from those observed in eukaryotic Ser/Thr or Tyr kinases and prokaryotic HKs. These domain arrangements unique to bacterial STYKs are therefore suggestive of their functional roles distinct from that of other well-known families of protein kinases. Consideration of known functions of domains tethered to catalytic kinase domain of bacterial STYKs enabled obtaining clues about gross functional roles of various bacterial STYKs. Involvement of STYKs in stress response, protein folding, extensive protein-protein interactions and sugar transport has been suggested by covalent tethering of catalytic kinase domains of STYKs with other enzyme and non-enzyme modules. Homologues of bacterial PKs that have been previously well characterised in terms of biochemical activity and function have also been identified in the study. Putative substrates for a small set of bacterial STYKs have also been identified based on their similarity to bacterial kinases with well characterised substrates.

## Results

The STYK-like sequences have been identified in various completely sequenced bacterial genomes and have been analysed for the occurrence of other functional domains. The domains have been identified using different profile search methods with stringent search parameters as described in the section on *Materials and Methods *and have also been manually checked for the conservation of key motifs. It should be noted that we have used stringent e-value cut-off of 10^-8 ^and 0.01 respectively in the PSSM (Position Specific Scoring Matrix) matching and HMM (Hidden Markov Model) matching procedures. All the functional domain assignments discussed below are detected within the e-value limit of 0.01 by HMMER and are ensured, manually, that there is no obvious erroneous domain assignment.

### Distribution of the STYKs and STYK-like sequences in bacterial genomes

The data set of bacterial genomes considered in the current analysis includes predicted ORFs from the genomes of sixteen archaeal and 109 bacterial genomes representing major division of these prokaryotic phylogenetic domains. A list of these completed genomes and number of Protein Kinase-like sequences (PKLS) that includes STYKs, RIO1 and ABC1-like kinases is given in Table 1. Table 1 also includes information on number of protein kinase-like sequences lacking the catalytic base residue (aspartate) and hence are likely to be non-functional as kinase. All the archaeal genomes analysed have at least one STYK, which belongs to the RIO1 family [19]. The RIO1s share a significant sequence similarity to the eukaryotic protein kinases and have recently been shown to possess a Ser/Thr autophosphorylation activity [35]. These RIO1 sequences have been initially observed in yeast [33,34] and subsequently detected in other eukaryotic genomes as well [36]. We have previously identified RIO1 like sequences in two of the eubacterial species, *Deinococcus radiodurans *and *Pseudomonas aeruginosa *[32].

Occurrence of RIO1 in many other eubacterial genomes has been reported in this paper for the first time. RIO1 sequences have been identified in other bacterial species including *Bradyrhizobium japonicum*, *Pseudomonas putida*, *Pseudomonas syringae*, *Shewanella oneidensis *and *Yersinia pestis*. The ubiquitous nature of RIO1 across archaea, bacteria and eukaryota is thus evident. Examination for the occurrences of HKs among the genome analysed reveal their absence in significant number of archaeal genomes, as well as in mycoplasma, *Buchnera *and *Onion yellows phytoplasma *genomes.

### Domain arrangements in bacterial homologues of eukaryotic protein kinases

Interactions of multi-modular signalling proteins with cognate ligands are often mediated by the constituent modules. The domain organisation of the STYKs has been analysed extensively by various profile-search and fold recognition methods described in a later section. The details of the domain organisation of the bacterial kinases analysed in this paper have been included in the KinG database [31,37]. Incorporation of information about STYKs in 125 prokaryotic genomes in the KinG database represents a major update in the database compared to the previous version with STYKs only from 35 prokaryotic genomes. A comprehensive study on the domain organisation of the previously compiled repertoires of STYKs and those encoded in the additional genomes has been described here. Modular organisation of STYKs, newly identified in the current analysis have been listed in Table 2

Influence of the diverse domain composition of STYKs and their probable biological functions are discussed in the following sections.

**Table 2:** Representative modular organisation of bacterial STYKs identified in the current analysis.

**Representative STYK**	**Domain organisation as identified in the current analysis**	Bacterial species encoding STYKs of similar domain organisation
gi|9947763	Phosphatase-2C (PP2C) + Kinase	*Pseudomonas aeruginosa, Pseuomonas putida. Deinococcus radiodurans.*
gi|32474490	Kinase + HSP70	*Pirellula sp.*
gi|31793271	Kinase + Hydrophobin	*Mycobacterium bovis*
gi|3261694	Kinase + MalT-like ABC transporter domain	*Mycobacterium tuberculosis H37Rv*
gi|41407147	Kinase + trans-membrane segment + peptidyl-prolyl cis-trans isomerase domain	Mycobacterium avium sub sp. paratuberculosis
gi|23020879	Kinase + 6 TPR repeats + GGDEF	*Clostridium thermocellum*
gi|17229994	Kinase + pectinesterase	*Nostoc sp. PCC 7120*
gi|17134087	Kinase + GUN4	Nostoc sp. PCC 7120
gi|9945898	Kinase + von Willebrand Type A domain	*Pseudomonas aeruginosa*
gi|17129689	Kinase + ANF Receptor	*Nostoc sp. PCC 7120*
gi|21223285	Kinase + bacterial extracellular solute binding protein	*Streptomyces coelicolor*
gi|1006577	Kinase + SH3b	*Synechocystis species*
gi|17129893|	FHA + Kinase	*Nostoc sp. PCC 7120, Thermodesmium erythraeum, Thermosynechococcuselongatus, Chloroflexus aurantia, Nostoc punctiforme.*
gi|1709642 (PKN2)	Kinase + Guanylate cyclase + Trans-membrane segment	*Myxococcus xanthus*

### Transmembrane bacterial Ser/Thr kinases

**Figure 1:**
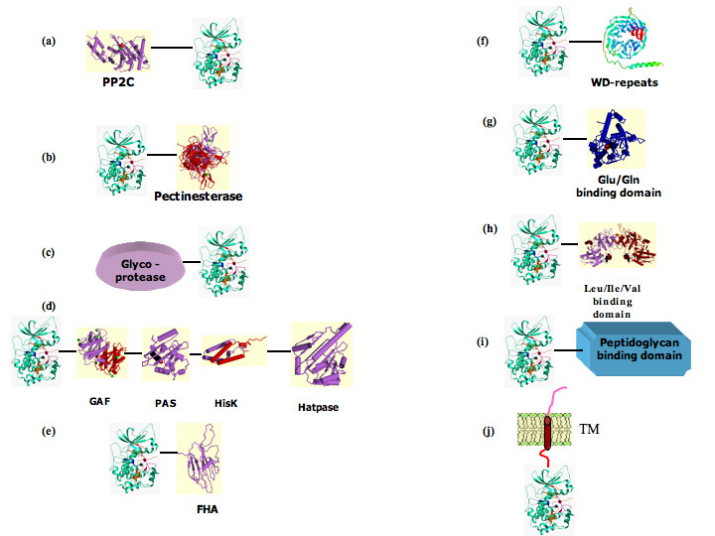
The representative domain combinations of the bacterial homologues of eukaryotic protein kinases are shown. The accession numbers of the protein domain families classified by PFAM are given below. (a) PP2c: phosphatase 2C [PF00481]; (b) Pectinesterase [PF01095]; (c) Glycoprotease [Peptidase_M22; PF00814]; (d) GAF[PF01590], PAS[PF00989], HisK[PF00512], HATPase[PF02518]. (e) FHA: Fork head associated domain [PF00498]; (f) WD-repeats [PF00040]; (g) and (h) amino acid binding domain [ANF_receptor; PF01094]; (i) peptidoglycan binding domain [PASTA, PF03973] k: trans-membrane segment [TM] shown in red.

The amino acid sequences of kinase catalytic domains of bacterial STYKs are generally more similar to eukaryotic Ser/Thr kinases than the eukaryotic tyrosine kinases. The profiles of Ser/Thr kinase subfamilies match the bacterial kinases with better similarity (<1e-32) in contrast to the tyrosine kinases (>1e-17). However a significant number of bacterial STYKs contain trans-membrane spanning segments unlike the eukaryotic Ser/Thr kinases. The known rare exceptions of eukaryotic transmembrane protein with Ser/Thr kinase domain are TGF-beta receptor and the IRE1. Trans-membrane STYKs are absent in certain Gram-negative species *Haemophilus influenzae*, *Rickettsiae*, *Helicobacter pylori*, *Chlamydiae *and in the hyperthermophilic bacteria, *Aquifex aeolicus*. The Gram-positive species analysed in the data set have at least one trans-membrane STYKs in them. The presence of significant number of trans-membrane kinases (TM-kinases) in most bacterial species suggests many of them could play as receptor like kinases and are probably involved in direct interaction with the extra-cellular ligands. Further the TM kinases with extracellular PASTA (Fig 1i) domain such as PKNB [28,29] of *Mycobacterium tuberculosis *are encoded in diverse groups of Gram-positive bacteria analysed in the current study. The selective conservation of PASTA domains in STYKs encoded by Gram-positive bacteria implies their role in signalling pathways conserved across diverse Gram positive species. Currently the nature of ligands stimulating the trans-membrane STYKs is not known.

### Archaeal multi-domain STYKs

The multidomain STYKs of archaea include the kinases with O-sialoglycoprotein endopeptidases (OSGP) domain (Fig: 1c) present at the N-terminal of the kinase domain. The OSGP domains belong to the glycoprotease family (PFAM00814). The members of this family are known to cleave proteins that are heavily sialylated on serine or threonine residues. The OSGP containing STYKs described in earlier studies [19] are also encoded only in archaeal genomes. The archaeal species with the OSGP domains in their STYKs include *Methanococcus janaschii*, gi|2826367), *Methanothermobacter thermoautotrophicus *(gi|2622538), *Halobacterium sp *(gi|1058146), and *Thermoplasma acidophilum *(gi|10639497). OSGP domains of archaeal STYKs bear highest sequence similarity with the OSGP domains of the yeast gene product YK18 which is a putative glyco protease.

### Phosphatase associated protein kinases

We report for the first time the tethering of the PP2C domains with STYK domain in bacteria. This unique class of STYKs has not been characterised so far. The PP2C like domains (Fig 1a) are tethered to the N-terminal of kinase domains in few STYKs encoded in *Pseudomonas putida *and *Pseudomonas syringae*. In addition to the well-characterised STYK of *P.aeruginosa *[38] a protein kinase (gi|9947763) with phosphatase-like domain described above has been identified in the current study. Earlier studies have biochemically characterised Stp1 of *Pseudomonas aeruginosa*, a single domain protein homologous to PP2C [38]. The regulation of Stp1 by its neighbouring gene encoding the protein kinase Stk1 has been suggested to be similar to the regulation of phosphatase and Ser/Thr kinases, YopH and YpkA of *Y.pseudotuberculosis *that serve as critical virulence determinants. Phosphatase like domain of Pseuodomonas species of the composite STYK shares 35% identity with Stp1 and about 30% identity with PP2C domains of Gram positive bacteria.

### Protein kinases associated with nitrate disimilation

A group of STYKs (gi| 7190465, gi|4376414, gi|8978521, gi|7189538) specifically conserved in different species of *Chlamydiae *has a highly conserved C-terminal domain. A domain family previously referred to as NirV in Conserved Domain Database [39] has been assigned to this C-terminal region (E-58). This homologous domain family comprises of NirV like proteins, methylase involved in chemotaxis and sulphatase modifying enzymes and is referred to as DUF323 (Domain of unknown Function) in the PFAM database. The crystal structure of DUF323 has been recently solved [40] for a human homolog of Formylglycine generating enzyme (pFGE), which is a non-functional counter part of human FGE. FGE modifies the active site residue glycine of the sulphatases. The NirV domain of the Chlamydial kinases shares a sequence identity of 37% over a stretch of 100 residues with the NirV protein from *Rhodobacter spaeroides*. The NirV gene is situated in the operon containing two other genes namely, NirK and ppaZ whose gene products regulate nitrate dissimilation. The disruption of NirV gene in other organisms does not affect the nitrate reduction [41].

### Protein kinases with domains homologous to modules associated with chaperones

We have identified a protein kinase (gi|32474490) with C-terminal domain homologous to HSP70 family of proteins in Pirellula species. The HSP70 family includes proteins that act as chaperones and assist the folding of a wide range of proteins.

Another class of STYKs that are also potentially involved in interactions with chaperonic proteins has been identified. STYKs with Tetratrico peptide repeats (TPR) have been identified in a number of bacterial species including *M.tuberculosis*, *Clostridium acetobutylicum*, *Gloeobacter violaceus*, *Leptospira interrogans*, *Nostoc sp*., *Pirellula*, *Streptomyces avermitilis*, *Sulfolobus solfataricus and Sulfolobus tokodaii*. TPR is known to form a series of anti parallel amphipathic α-helices that bundle together through hydrophobic interactions to form a fold with deep concave groove. They are suggested to bind a number of proteins of regulatory importance such as HSP90 based chaperone system [42].

### Stress-responsive protein kinases

In the current analysis we have identified STYKs (gi| 13475495, gi|27353958 and gi|30180007) with universal stress induced protein associated (USPA) domain encoded in the genomes of *Mesorhizobium loti*, *Bradyrhizobium japonicum *and *Nitrosomonas_europaea*. Protein kinases with USPA domains have been recently identified in STYKs of *Arabidopsis thaliana *[43] and HisK of bacteria [44].

### Protein kinases with protein – protein interaction modules

Protein kinases considered in the analysis have also revealed their association with domains made up of repeats that serve as an interface for the interaction of protein modules. An other class of STYKs with PQQ family repeats have been identified in *Deinococcus radiodurans*, *Streptomyces avermilitis *and *Streptomyces coelicolor *that have been described in the greater detail in later section with respect to their role in regulation of secondary metabolism. The PQQ repeats are known to form β-propeller like structures similar to WD – repeats that could serve as sites of interactions with target proteins.

STYK (gi|9948555) with leucine-rich repeats (LRR) have also been identified in *Pseudomonas aeruginosa*. LRRs are known to serve as protein interaction domains that form non-globular structures made up repeated ba subunits [45].

NHL repeats are also identified in two STYKs of *M. tuberculosis *(gi|2078052) and *D.radiodurans *(gi|6457717) which have 6 and 3 repeats following the kinase domain. They are known to serve as protein interaction surfaces and some times possess enzymatic activity as exemplified by the bifunctional peptidyl glycine α-amidylating monoxygenase protein.

### Ubiquinone biosynthesis associated protein kinases

Significant number of archaeal and bacterial genomes encode STYKs with regions most similar to ubiB gene products flanking the catalytic kinase domain. Ubiquinone, is a critical component of the respiratory chain of most of organisms. The gene product of ubiB is involved in the production of an intermediate of Ubiquinone or the co-enzyme Q [46]. Based on the sequence comparisons, this group of kinases has been predicted to be involved in the regulation of ubiquinone biosynthesis [19]. This group of STYKs had been grouped into ABC1 family and was initially believed to have restricted occurrence in archaea [19]. The currently available genomic data reveals a wider representation of the ABC1 kinases in significant number of archael species.

They include *Halobacterium salinarium, Thermoplasma acidophilum, Methanosarcina acetovirans, Methanosarcina mazei, Sulfolobus sulfotaricus, Sulfolobus tokadii, Thermoplasma volcanium *All the ABC1 kinases encoded in archeal species analysed in the current study have an N-terminal ABC1 kinase domain and a putative trans-membrane spanning segments at the C_terminal. *Thermoplasma acidophilum (gi|10639567) *also has an unique ABC1 kinase with N-terminal RI01 like domain and C-terminal ABC1 Kinase-like domain followed by a putative trans-membrane spanning segment. ABC1 family of STYKs has a wide representation in large number of bacterial species. As described before *Nitrosomonas europaea *encodes a STYK of ABC1 family that has a N-terminal Usp domain.

### Phosphorylation and structural components of bacterial cells

A set of closely related protein kinases is conserved in *Mycobacterium bovis subsp. bovis AF2*, *Mycobacterium tuberculosis CDC1551 *and *Mycobacterium tuberculosis H37Rv*. This new group of kinases identified in the current analysis has a N-terminal protein kinase domain and a C-terminal hydophobin like regions. The hydrophobins are low molecular weight cysteine rich proteins that form a critical component of the hydrophobic sheaths covering the fungal spores.

STYKs conserved among all the Gram-positive bacteria considered in the current analysis, have a C-terminal region that is highly similar to the C-terminal region of high molecular weight penicillin binding proteins. This domain is believed to play a role in the recognition of D-alanyl D-alanine dipeptides used to build up the peptidoglycan layers and is classified into the family called PASTA [47] in Pfam database due to their occurrence in bacterial Ser/Thr protein kinases and penicillin binding proteins.

### Other domain combinations observed in bacterial protein kinases

A STYK found in the *M.tuberculosis *(gi|3261694) is comprised of an N-terminal kinase domain followed by C-terminal domain that shares high sequence similarity to distinct group of ABC transporters, MalT (E-value:10^-180^). MalT group of transporters are unique in terms of their direct participation of the transporter in transcriptional regulation of *mal *regulon [48]. These observations suggest the possible role of the STYK in the regulation of uptake of sugars. This domain has been observed only in *M.tuberculosis *STYK and is not found in the closely related species, *M.leprae*. This protein kinase is likely to be critical for nutrient uptake.

*M.tuberculosis *also contains a PK (gi|2131007) with a domain that shares significant similarity (e-value 10^-4^) with the periplasmic oxido reductase of thioredoxin family (DSBA). The thioredoxins are known for their interaction with a broad range of protein as a general protein disulphide oxido reductase. The potential role of this kinase for its possible involvement in disulphide isomerisation of various proteins during protein folding is therefore suggested. The occurrence of the DSBA domain in the extracellular region of the PK emphasises their role in reduction of periplasmic mycobacterial proteins. This is unique to *Mycobacterium tuberculosis *and is not found in *M.leprae*.

The genome of *Mycobacterium avium subsp. paratuberculosis *also encodes another STYK probably assisting the folding of proteins. The domain orgainisation of this novel kinase includes an N-terminal kinase domain, a central transmembrane domain and a C-terminal peptidyl-prolyl cis-trans isomerase (Pro_isomerase) domain. Among all available completely sequenced genomes of various species of Mycobacteria, this novel protein kinase is encoded in only in the *M.avium *genome.

*Clostridium thermocellum *ATCC 27405 encodes one of the largest STYK of 1826 amino acids encompassing a N-terminal kinase domain with six TPR repeats followed by the GGDEF domain at the C-terminal end. The domains of sensor kinases and response regulators of the His-Asp phospho relay systems have been studied previously [49]. The GGDEF domain is suggested and subsequently shown to be homologous to the catalytic domain of adenylyl cyclases [50,51]. Recent studies have also suggested their role in the regulation of bacterial growth and development through novel response regulators [52] by turnover of cyclic diguanosine monophosphate. Studies have also underlined the importance of adjacent sensor domains in the regulation of the catalytic activity of GGDEF [53]. The occurrence of GGDEF with STYKs implies the sharing of down stream signalling components by the HisKs and STYKs and their role in production of cyclicdiguanylate.

The Nostoc sp. PCC 7120 encodes a protein kinase (gi|17229994) with a pectinesterase domain (Fig 1b). The pectin esterases catalyse the hydrolysis of pectin, a component of extra cellular capsules and cell walls. The association of two such enzymatic activities is unique to this organism and suggests the probable role of phosphorylation in maintenance of integrity of bacterial cellular structural components.

A novel protein kinase (gi|17134087) with a C-terminal GUN4 domain has been identified in the Nostoc sp. PCC 7120. The GUN4 proteins in *Arabidopsis thaliana *are known to bind to magnesium-protoporphyrin IX, an intermediate in chlorophyll biosynthesis and there by regulating the enzyme Mg-chelatase involved in the biosynthesis [54]. The novel protein kinase is therefore suggested to be involved in the regulation of chlorophyll production in Nostoc species.

*Pseudomonas aeruginosa*, known to be an opportunistic pathogen encodes a total of 11 STYK-like sequences. Seven of these are STYKs and the rest are KDO kinases. We have identified the von Willebrand A domain located to the C-terminal of kinase domain in one of the STYKs that has been previously referred to as Ppka [55]. The glycoprotein binding vWA domain [56] might be playing an important role in its interaction with the host proteins as described in the later sections.

A STYK (gi|3328716) in *Chlamydia *species has a N-terminal kinase domain, and a C-terminal domain with similarity to TPR domain. Using remote homology detection methods as described in the 'Materials and Methods' section, the similarity of this region with the TPR domain of O-linked N-acetylglucosamine (O-GlcNAc) transferases [57] has been detected. The TPR domain of O-GlcNAc transferase (OGT) has been recently shown to be responsible for its targeting OGT and in determining its substrate specificity [58]. The domain homologous to TPR in the STYK is therefore likely to mediate its substrate interactions.

### Ser/Thr kinases of bacteria with periplasmic protein domains

The periplasmic solute binding proteins are a part of the ATP binding cassette (ABC) transporter that transfers the amino acids and sugars into the cytoplasm. Ser/Thr kinases from *Streptomyces coelicolor *(gi|21223285) and Nostoc sp. 7120 (gi|17227840|) contain a solute binding domain for Glu, Gln (Fig 1g) and ANF receptor like domain (Fig 1h), which is involved in the binding of branched chain amino acids respectively.

### Bacterial STYKs with non catalytic domains homologous to domains associated to eukaryotic PKs

This section describes the possible functions of a few bacterial STYKs inferred based on their similarity in domain combination with the well characterised eukaryotic protein kinases.

*Synechocystis sp*. is a cyanobacteria that encodes 12 STYKs. One of the STYK (gi|1006577) has a SH3b (bacterial SH3 domain) following the N-terminal kinase domain. Occurrences of SH3 domains in bacteria have been previously well documented [59]. They have also been classified into a different family in SMART database [60].

Protein kinases with WD repeats (Fig 1f) were identified in many bacterial species including *Thermospora curvatum*, *Thermodesmium erythraeum*, *Chloroflexus aurantia*, *Nostoc punctiforme*, *Nostoc sp*. PCC 7120 and *Thermobifida fusca*. Most of these kinases contain 7 WD repeats and are identified in cyanobacteria. The number of repeats varied in few from 4 to 14 and all the species mentioned above contain more than one such protein kinase with the exception of *Thermobifida fusca*. The WD repeats are associated with beta-propeller like folds and the most extensively studied are the Gβ-subunit of the heterotrimeric G-proteins in eukaryotes.

**Figure 2:**
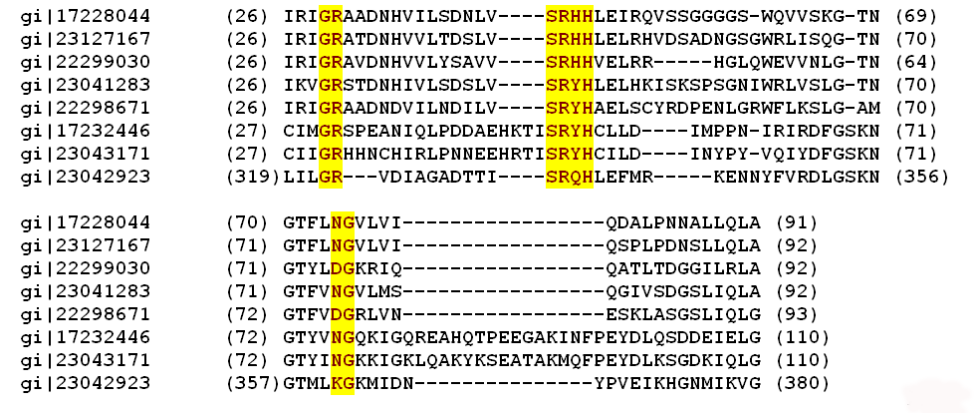
Multiple sequence alignment of FHA domains seen in bacterial Ser/Thr kinases with the conserved motifs involved in phospho-threonine binding, 'GR', 'SXXH', 'NG' shaded in yellow. gi|17228044|ref|NP_484592.1| (*Nostoc sp. PCC 7120*), gi|23127167|gb|ZP_00109042.1| (*Nostocpunctiforme*), gi|22299030|ref|NP_682277.1| (*Thermosynechococcus elongatus BP-1*), gi|23041283|gb|ZP_00072750.1| (*Trichodesmium erythraeum IMS101*), gi|22298671|ref|NP_681918.1| (*Thermosynechococcus elongatus BP-1*) gi|17232446|ref|NP_488994.1| (*Nostoc sp. PCC 7120*) gi|23043171|gb|ZP_00074493.1| (*Trichodesmium erythraeum IMS101*) gi|23042923|gb|ZP_00074272.1| (*Trichodesmium erythraeum IMS101*).

The FHA domain containing PKs (Fig: 1e) have been identified in the current analysis in the cyanobacterial species, *Thermodesmium erythraeum *(gi|23042923, gi|23043171, gi|23041283), *Thermosynechococcus elongatus *BP-1 (gi|22298671, gi|22299030|) *Chloroflexus aurantia *(gi|22970578), *Nostoc punctiforme *(gi|23127167), and *Nostoc sp*. PCC 7120 (gi|17232446, gi|17228044, gi|23043171). The phospho – threonine binding resiudes G**R**, **S**xx**H **and **N**G are well conserved in all these FHA domain containing protein kinases (Fig. 2). In eukaryotes the FHA containing protein kinases are conserved from yeast to mammals and are involved in the regulation of the various stages of cell cycle and development [61].

**Figure 3:**
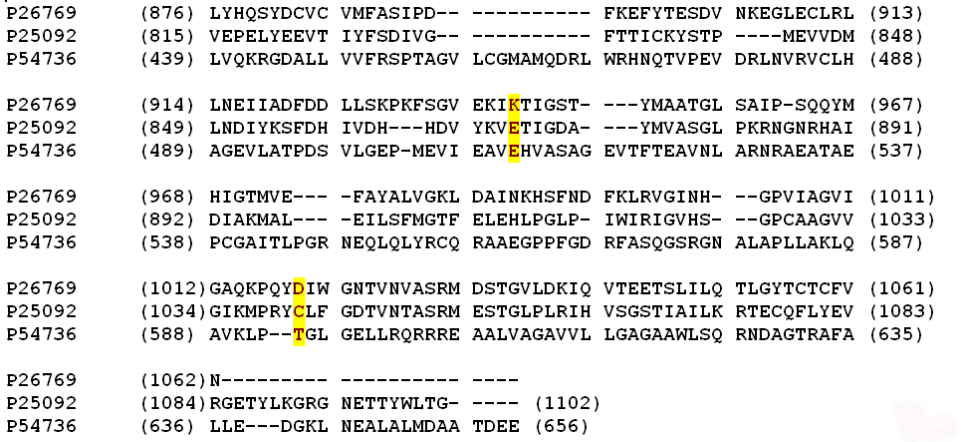
Multiple sequence alignment of the human guanylate cyclase, rat adenylate cyclase and GC-like domain of PKN2 of Myxococcus xanthus is shown. The residues conferring nucleotide specificity are shaded yellow. Conserved hydrophobic and charged residues are shaded green and grey respectively.

The *Myxococcus xanthus *protein kinase, PKN2 has an interesting domain organisation. The PK consists of an N-terminal kinase domain that extends as a stretch of Ala/Ser/Pro/Thr rich segments which further continues as a trans membrane segment as described in earlier studies [62,63]. We have identified a Adenylyl/ Guanylyl cyclase (AC/GC) domain in the region between the Ala/Ser/Pro/Thr rich regions and the trans membrane (TM)- region. Regions homologous to cyclases have been also observed in previous studies [64,65]. Cyclasess of this nature also occur in the eukaryotes. A stretch of nearly 200 residues with no similarity to any extra cellular domains known so far lies on the external side of the TM segment. However the protein kinase like domain (PKLD) in the eukaryotic GCs lack the catalytic residues. In the case of PKN2 the PK domain has all the catalytic residues and is also known to phosphorylate on Ser/Thr residues [62]. The conservation of catalytic residues of cyclase domain and the topology of the gene product has been analysed in the current study. The key residues at the catalytic sites of the GC, Glu and Cys confer nucleotide specificity to the enzyme. Mutation of Glu and Cys to Lys and Asp, the corresponding residues of the adenylyl cylases, changes the nucleotide specificity of GC to adenylate [66]. Among the residues known to confer the nucleotide specificity of the GCs the glutamate residue is conserved (Fig. 3) while the other residue involved in specific nucleotide binding, Cys, is substituted by Thr which is found in equivalent positions in few the adenylate cyclases. The nucleotide specificity of the GC-like domain of PKN2 sharing less than 15% identity with receptor GC is unclear. Trans membrane region of PKN2 follows the GC domain unlike eukaryotic transmembrane GCs.

A wide representation of eukaryotic signalling domains in the bacterial kingdom has been described in previous studies [67-69] and is also manifested in the bacterial Ser/Thr kinase family. Such domain recruitments across diverse signalling protein families imply the versatility in the nature of interactions of these domains.

### Dual protein kinases in bacteria

It has been observed in the yeast, an unicellular eukaryote, that the osmo – regulation is brought about by both HisK and mitogen activated protein kinases (MAPKs) suggesting the signal transduction from HisK to the MAPKs to elicit the appropriate response [70,71]. The occurrences of such composite signalling pathways have been suggested in bacteria from other very early studies [72] and in studies on STYKs in *Anabaena sp*. PCC 7120 [72]. The STYKs with HisK, HATPase (the catalytic domain of HisKs) and GAF (regulatory domains found in phosphodiesterases, adenylate cyclases and pytochromes) have been referred to as dual protein kinases (Fig 1d) as they contain catalytic domain of two distinct kinases fused to form a single gene product. Hybrid kinases such as those described above have so far been identified in cyanobacterial species [64,65,73,74]. The cyanobacterial species *Nostoc sp*. PCC 7120 and *Nostoc punctiforme *have 12 and 11 dual protein kinases respectively. In the current study, the occurrences of such composite kinases have been noted in 5 different species. The other organisms that encode similar STYKs include *Trichodesmium erythraeum*, *Leptospira interrogans, Ralstonia metallidurans *and *Rhodopsuedomonas plustris *and *Psuedomonas putida*. The nature of signals these composite kinases perceive and the cellular processes they influence is intriguing. Some of these STYKs also have the PAS domains in the same polypeptide chain which is likely to serve as redox sensors and thereby suggesting the roles of such dual protein kinases in signalling pathways sensitive to redox potentials.

### Protein kinases involved in bacterial cell division

Protein kinases encoded in the genomes of *Bacillus subtilis *and *Bacillus halodurans*, yabT (gi|2632333gnlPIDe1181999) and BH0080 (gi|10172692dbjBAB03799.1) respectively, are located in the same gene clusters as *spoIIE *and *FtsH/MesJ*. These STYKs are therefore suggested to be involved in the control of cell division like other genes in the same cluster. Among the many genes known so far to control the cell division in bacteria are the *spoIIE *and the *FtsH*/*MesJ *genes [75]. This group of STYK that are likely to be associated with cell division are analogous in their broader function to cyclin dependent kinases of the eukaryotes that control cell cycle.

### Bacterial protein kinases and their substrates

Despite the identification of bacterial STYKs by genome-wide analysis and demonstration of *in vitro *phosphorylation activity on exogenous substrates of significant number of STYKs, very limited information is available regarding their natural substrates and their specificity.

Putative homologues of AfsK, a well characterised Ser/Thr kinase *S.coelicolor*, has been identified in *Deinococcus radiodurans *and *Streptomyces avermitilis*. In *S. coelicolor*, AfsK phosphorylates of a global regulator of secondary metabolism AfsR that also controls the production of Actinorhodin [17,76,77]. This is brought about by the Ser/Thr kinase AfsK. AfsK, encoded in the genomes of *Streptomyces coelicolor *and *S. lividans *is a membrane associated phosphokinase encoded by the afsK gene. The analysis of the domain composition of AfsK revealed the presence of nine bac_PQQ repeats located at the C-terminal of the protein.

A search for the AfsR homologues in *Deinococcus radiodurans *that are likely to be the putative targets of Afsk revealed a gene product (gi|15807540) with N-terminal transcription regulator domain (trans_reg) and Bacterial trans-activation domain (BTAD) similar to the domains observed in AfSR. This observation suggests that the gene product could probably be the target of the Afsk like kinase (gi|6460339) of *D.radiodurans*. Trans_reg or the BTAD domain conserved among all the transcription regulators of AFSR family are therefore likely to harbour sites for phosphorylation by AfsK.

The STYK sharing highest sequence similarity with AfsK in the catalytic domain (38% identity) in the *M.tuberculosis *genome is PKNB. A search for *AfsR *like gene products in the *M.tuberculosis *genome revealed the gene product of *embR *as its closest homologue. A further sequence analysis of embR gene product is known to be involved in the resistance to etambutol revealed the presence of BTAD, transactivation domain. This common domain shared by the AfsR and its homologue in *M.tuberculosis *and *D.radiodurans *further suggests that the BTAD, transactivation domain is critical for regulation of AfsR like gene products and as the most likely region of the phosphorylation mediated control of its activity. Recently embR gene product has been demonstrated to be phosphorylated by PKN-H [78].

Substrate identification studies on PKN2 of *Myxococcus xanthus *have revealed histone like proteins (HUα and HUβ)[63] and beta-lactamase [79] as the target proteins phosphorylated by the recombinant PKN2 expressed in *E.coli*. The phosphorylation of beta-lactamase inhibits its secretion [79] while the phosphorylation of HUα and HUβ+ [63] has been shown to prevent their binding to DNA resulting in toxicity to cells. PKN4 has been further been shown to phosphorylate phosphofructo kinase [80,81].

### Phosphorylation as a mode of regulation of bacterial STYKs

**Figure 4:**
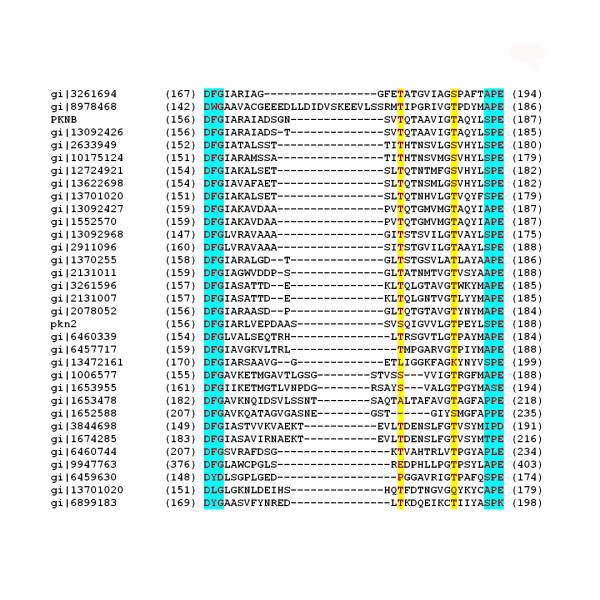
Multiple sequence alignment of the activation segment of 'RD' protein kinases in bacteria with the canonical 'DFG', 'APE' shaded in blue and conserved threonines, the potential autophosphorylation sites shaded yellow. gi2911096, gi1370255, gi2131011, gi3261596, gi2131007, gi2078052, (*M.tuberculosis*); gi13092426 gi13092427 gi13092968 (*M.leprae*); gi8978468 (*C.pneumoniae*); gi2633949 (*B.subtilis*); gi10175124 (*B.halodurans*); gi12724921 (*Lactococcus lactis*); gi13622698 (*S.pyogenes*); gi13701020 gi13701020 (*S.aureus*); gi6460339 gi6457717 gi6460744 gi6459630 (*D.radiodurans*); gi13472161 (*M.melitoti*); gi1652313, gi1006577 gi1653955 gi1653478 gi1652588 (*Synechocystis.sp*); gi3844698 (*M.genitalium*); gi1674285 (*M.pneumoniae*); gi9947763 (*P.aeruginosa*); gi6899183 (*U.ureolyticum*).

Among the completed genomes analysed in this study, a significant number of STYKs are 'RD' protein kinases. The protein kinases in the eukaryotes are in turn regulated in a number of ways. Phosphorylation of Ser/Thr residues in the activation segment of the catalytic domains of these kinases is a common mode of regulation [82]. The PKs that undergo phosphorylation in the activation segment have a characteristic 'RD' doublet sequence motif in the catalytic loop wherein the Arg helps in neutralisation of the negative charge on the phosphorylated residue to suitably poise the catalytic aspartate for phosphorylation. These protein kinases are referred to as 'RD' kinases. A multiple sequence alignment of the putative activation segment in the catalytic domains of the bacterial 'RD' kinases have revealed the strong conservation of threonines in their activation segments (Fig: 4) These threonines are therefore suggested to be the sites of phosphorylation. Most of these kinases have two threonines that are well conserved and hence multiple phosphorylation for activation of these kinases as exemplified by the PKN-B of *M.tuberculosis *[83] is suggested.

## Discussion

The genome-wide analyses reveal the occurrences of a large number of STYKs in bacteria. However the available knowledge about their biological functions is limited.

The STYKs show a lower representation compared to HKs in most of the genomes analysed (Data not shown). HKs are encoded by higher number of genes in comparison with STYK encoding genes in the prokaryotes. Nearly two percent of the genomes of eukaryotic organisms encodes STYKs as suggested from the genome-wide analysis by various groups [34,84-87]. Difference in the relative abundance of kinases belonging to the two classes, namely HKs and STYKs in the eukaryotes and the prokaryotes probably arises from the selection of specific class of PKs during the course of evolution, as an adaptation to distinct cellular environment. Low occurrence of STYKs compared to HKs in most bacterial species with the exception of Pirellula (Rhodopirellula baltica SH1) suggests, in general, the predominant role of HKs in prokaryotes.

HisK in archaeal genomes have been suggested to have arisen from lateral gene transfer events explaining their limited representation [88,89]. With the exception of *Buchnera, Onion phytoplasma *and *Thermococcus kodakaraensis*, all the above mentioned genomes have at least one of the two kinds of protein kinases, namely HisK and STYK. STYKs have not been identified in genomes of the two spirochaetes, *Treponema pallidum *and *Borrelia burgdorferi, Chlorobium tepidum, Helicobacter hepaticus, Thermotoga maritima *and *Xanthomonas species*. The absence of either classes of protein kinases in *Buchnera *species, an obligate symbiont of the Aphids and *Onion phytoplasma *(intra-cellular plant pathogen) suggests the loss of such enzymes in the above species is likely to be an adaptation to the host-intracellular environment. Our analysis also identifies an anaerobic obligate heterotrophic thermophile, *T.kodakaraensis *as the only species with a non-symbiotic or non-parasitic habitat that lacks HisK and STYKs. Further studies on influence of phosphorylation on response to external stimuli will therefore be able to provide insight into the nature of enzymes and significance of phosphorylation in these organsims.

The number of STYKs in phylogenetic domains of archaea and bacteria increase with the growing size of bacterial genomic data. The assignment of probable biochemical functions and biological roles to large number of sequences are often dependent on the occurrence of homologous gene products in other phylogenetic domains despite the vast divergence between the species. Earlier studies have also indicated that the gross biological function and interaction network of multi-domain proteins are conserved by the preservation of domain composition and arrangement [90-92]. Therefore domain organisation of the bacterial STYKs have been studied here to get an insight into their putative biological roles in comparison with other proteins of similar domain organisation.

Enzymatic and non-enzymatic domains described in the earlier sections have been found covalently tethered to the kinase catalytic domain. Among the archaeal multi-domain STYKs, the OSGP-containing STYKs are widely represented. Knowledge of substrates of the OSGP-containing STYKs and their sites of phosphorylation is required to understand their role in regulation of functions of glycoproteins. The Ser/Thr residues serve as sites for both glycosylation and the phosphorylation [93,94]. In many instances the phosphorylation and glycosylation occurs at common sites in a competitive manner. The phosphorylation would therefore prevent the glycosylation of the proteins. OSGP domains are involved in the cleavage of those proteins at the sites containing glycosylated Ser/Thr residues. The phosphorylation of the target proteins by the associated kinase domains of the OSGP containing STYKs might therefore serve as a regulatory mechanism to inhibit the proteolysis of glycoproteins.

Eubacterial STYKs are associated with a large variety of signalling domains. Chase 2 domains containing STYKs identified by recent studies [64,65,73] are also found in other classes of trans membrane receptors including histidine kinases, diguanylate cyclases and methyl accepting proteins. Presence of widely occurring sensory domain in Ser/Thr kinases of *Nostoc *further emphasises on its role in the perception of some critical signals that are likely to trigger cellular pathways synergistically.

STYK of *P.aeruginosa *with phosphatase like domain has significant similarity with spoIIE protein family in the PFAM database. Recent studies [95,96] have shown the involvement of Ser/Thr kinase-Phosphatase pair, PrkC and PrpC in the sporulation of *B.subtilis*. *Psuedomonas aeruginosa *however encodes a composite kinase-phosphatase in the same gene. The phosphatase-2C (PP2C) domains are known to selectively dephosphorylate protein phosphorylated on Ser/Thr residues and their homologues have been previously identified and characterised in various bacteria [97-101]. The PP2C domains have been predicted to be functionally active in SpoIIE [102,103] of *Bacillus subtilis*, a Gram positive bacterial species. SpoIIE controls the activation of sporulation transcription factors and critical for the development of the bacteria.

The bacterial STYKs with TPR repeats identified here are suggested to be influenced by the chaperone like protein during their trafficking, folding or interactions with substrate proteins. The involvement of HSP90 in hormonal and growth factor signal transduction mediated by protein kinases through proteins containing TPR repeats is well characterised in eukaryotic cells [42]. Further phosphatase pp5 of *Plasmodium falciparum *with TPR domain is known to act as co-chaperone for hsp90 and it has been therefore suggested that protein dephosphorylation might play a regulatory role in protein folding [104,105]. Despite their high sequence divergence the TPR repeats and their constituting domain form a general interface of interactions with HSP90 like chaperones [104].

STYKs with stress response domains such as USPA have been identified. The USPA proteins are suggested to be autophosphorylated to switch on to active state. Subsequent to the induction of USPA proteins, many phosphorylated proteins have been detected [106]. The two-component system involved in nitrate assimilation is known to induce bacterial universal stress proteins of the nucleotide binding class [44]. Majority of these proteins are tightly regulated and expressed under various conditions of stress in the bacteria.

The USPA containing kinases of plants have been implicated in disease resistance signalling pathway based on their similarity to other protein kinases like Pto and Pto interacting proteins of tomatoes that are involved in disease resistance [43,107]. Based on the role of USPA containing proteins in stress response and their regulation by the some two component system, we suggest the bacterial STYKs with USPA domains are likely to be induced during stress response. In the eukaryotes, MAP kinases are also known to trigger stress induced signalling pathways. This group of bacterial kinases could therefore be functionally analogous to their eukaryotic counterparts that are induced during stress.

Protein kinase containing gene products with C-terminal pentapeptide repeats have been identified initially by an early study [73] in *Nostoc sp. PCC 7120 *(gi|17132363) and *Synechocystis sp. PCC 7803 *(gi|1653478). Nitrate assimilation and nitrogen fixation are well characterised in various species of Cyanobacteria. The key enzyme involved in this pathway, the nitrogenase, is highly sensitive to oxygen. Some multi-cellular cyanobacterial species like *Nostoc sp*. form specialised cells called heterocysts wherein the glycolipid layer prevents the diffusion of oxygen [77]. The accumulation of glycolipids into the heterocysts is brought about by a number of proteins. Some of these proteins have the pentapeptide repeats, which are known to be critical for the localisation of glycolipids [108].

PASTA domains conserved in a group of STYKs encoded by Gram positive bacteria are found in proteins comprising the divisome complex. The representation of this group of STYKs in all gram positive bacteria, including the *Lactococcus lactis *(gi|12724921), and Streptococcus pyogenes (gi|13622698), which contain only one STYK suggest their role to be critical in their cell division. The role of these protein kinases in cell division is reiterated with their genes clustering with *rodA *involved in the regulation of cell morphology during cell division. The phosphatase 2C encoding genes also lie in the same cluster supporting the role of reversible phophorylation during cell division.

Lipopolysaccharide kinases (LPSK) engaged in the phopshorylation of the lipopolysaccharides in the outer membrane of Gram-negative bacteria share remote similarity with eukaryotic STYKs [32]. They could be grouped into two classes based on their sites of phosphorylation into *waaP *gene products and Kdo kinases (KdoK) [109,110]. A remote similarity between STYKs and the two family of LPSKs has been recently reported by our group [32]. The similarity in the potential 3-D structure and catalytic residues of LPSKs with the eukaryotic protein kinases suggests analogous catalytic functions to the residues that are well conserved across the two distinct families of enzymes. The occurrence of Kdo kinase is so far restricted to pathogenic Gram-negative species and is also known to be influencing the virulence in *H.influenzae *[109]. Inferences drawn on the structure and the catalytic mechanism of LPSK based on their similarity to eukaryotic protein kinases can therefore serve as a guide to the design of inhibitors to the LPSKs in virulent organisms.

The genomic organisation of all STYKs encoded in *M.tuberculosis *H37Rv genome has been analysed in an earlier study [111]. The gene encoding the protein kinase PKN-G of *M.tuberculosis *is clustered with the genes, *glnH *for periplasmic Gln binding proteins. Therefore PKN-G is likely to be the functional homologue of the other Gln binding periplasmic Ser/Thr protein kinase of *Streptomyces coelicolor *(gi|21223285). These protein kinases therefore suggests the intake of amino acids in to the cell is also likely to be influenced by phosphorylation, in addition to the sugar transport in *M.tuberculosis *as described previously. Recent studies have established the involvement of PKN-G in the regulation of cellular levels of Gln/Glu [112,113] and in evading phagocytosis by the host cells.

Eukaryotic signalling domains have been observed in a large number of bacterial proteins including in the STYKs [67-69]. The cyanobacterial STYK with a SH3b domain has a transmembrane segment between the kinase domain and the SH3 domain. The nature of signals perceived by this trans-membrane STYK is not clear. These SH3 domains are distantly related to their eukaryotic counterparts. Eukaryotic Ser/Thr kinases with SH3 domains have been well studied which is exemplified by mitogen activated protein kinase kinase [114,115].

WD-repeats, a versatile protein-protein interaction module has also been observed bacterial protein kinases [64,73,116,117] and in human protein kinases [46]. Pkwa of Themospora curvata, a member of WD-repeat kinase family has been shown to be phosphorylated by exogenous protein kinase form *S. granticolor *[100].

The fork head associated (FHA) domain was initially found in transcription factors and has been recently identified as protein – protein interaction modules known to bind to phospho-threonine residues on target proteins. Hence the interactions of the FHA domains with their cognate proteins are largely influenced by phosphorylation. Many bacterial proteins have been recently identified with FHA domains [117]. Previously it has been observed that the genes encoding FHA containing proteins cluster along with Ser/Thr protein kinases and phosphatases in the *M. tuberculosis *genome [111]. Thus the identification of the PKs with FHA domains in cyanobacteria suggests synergistic actions of FHA domains containing proteins and enzymes involved in reversible phosphorylation as observed in eukaryotes.

Very few studies have shed light on the nature of substrates of bacterial kinase [17]. The AfsR, the substrate of AfsK has an N-terminal transcription regulator domain (trans_reg domain), trans-activation domain (bacterial trans-activation domain, BTAD) followed by a signalling domain NB-ARC and a C-terminal tetratrico peptide repeat region which is known to be involved in protein-protein interactions. The BTAD domain is shared by all the members DNRI/REDD/AFSR family of transcription regulators involved in secondary metabolism. Other proteins in *Streptomyces coelicolor *and related species that control actinorhodin production share the trans_reg domain and the BTAD domain. A similar domain organisation among the homologues of AFSR is intriguing and is likely to contain sites of phosphorylation.

The phosphorylation site of HUα has been identified as a threonine residue which is well conserved across HUα of various species. RXXR**T**GR is the sequence pattern conserved at the phosphorylation site (P-site). The occurrence of R at the P-1 site suggests PKN2 to be a 'basic directed' protein kinase like Protein Kinase A (PKA) that specifically phosphorylates substrates with consensus motif RRXS/TZ' (X is any amino acid and Z is any hydrophobic amino acid). Based on the residue preferences of PKN2, at known phosphporylation sites on substrates, bacterial STYKs sharing significant similarity with PKN2 have been examined, that are likely to seek similar residue determinants on their substrates. The catalytic domain of PKN2 shares highest similarity (40% identity) with PKNB of *M.tuberculosis *and its homologue in *M.leprae*. The high sequence similarity between PKN2 and PKNB is likely to reflect in similar substrate binding modes such as preferences for N-terminal basic amino acid residues as also observed for PKA.

PKN2 of *Myxococcus xanthus *is known to autophosphorylate in the region following the kinase domain. The GC domain that lies C-terminal to the kinase domain contains 10 threonines. Further experimental studies are therefore necessary to ascertain if these sites could be the natural phosphorylation sites targeted by their respective enzymes.

A significant number of bacterial STYKs have been biochemically characterised previously to elucidate their biological roles. STYKs of the PKN subfamily have been identified and well characterised in *Myxococcus xanthus *[79-81]*Streptomycetes coelicolor *[17], *Strepyomycetes granticolor *[86]*, Synechocystis sp *[118-120]*, Yersinia tuberculosis *[33]*, Mycobacterium tuberculosis *[111,122] and *Psuedomonas aeruginosa *[55]. The PKN representatives of *Myxococcus xanthus *and Streptomyces species are involved in the specialised developmental cycles characteristic to these species. They are also shown to be capable of autophosphorylation although it has been suggested to be unimportant for their kinase activity *in vitro *[17,79-82].

The YpkA of *Yersinia pseudotuberculosis *is a secreted STYK that has been previously shown to interact with small GTPases like RhoA and Rac1 of the host [26]. We have identified the homologue of YpkA, the only STYK in the completely sequenced genome of *Yersinia pestis *CO92 sharing 99% sequence identity with YpkA. However the genome of *Yersinia pestis *KIM does not encode any YpkA homologues. In addition to ABC1 and RIO1 class of STYKs, a single gene product containing only the kinase domain is identified.

PpkA of *Pseudomonas aeruginosa *have been implicated in their virulence and are suggested to be induced during the process of infection [55]. An unusual domain arrangement of PpkA identified in the current analysis comprises of an N-terminal kinase domain followed by a stretch of 300 residues of unknown function and a C-terminal von Willebrand factor Type A (vWA) domain. The vWA domain, known to bind to glycoproteins and growth factor receptors, in PpkA is therefore suggested to aid in interactions at the surface of the host cell.

Mbk, is a Ser/Thr kinase from *Mycobacterium tuberculosis *encoded by the gene mbk [121] that lies in pst operon that controls phosphate transport. Mbk corresponds to STYK gi|2078052 in *M.tuberculosis *genome, which has an N-terminal kinase domain, a transmembrane region and a C-terminal NHL-repeats. The occurrence of membrane spanning region and extra-cellular NHL-repeats that forms an interaction surface suggests the Mbk to serve as receptors for unknown ligands regulating the phosphate transport.

The STYK, referred to as SpkA in *Synechocystis *is previously shown to be required for the motility of the cyanobacteria. SpkA corresponds to STYK (gi|1652312), in the genome of *Synechocystis sp *that has an N-terminal kinase domain and a C-terminal extension of 200 residues to which no known functional domain could be associated. The role of the C-terminal extension in the regulation or interaction with other proteins controlling the motility of the organism remains to be elucidated

The attempt made in the current analysis to study the bacterial STYKs by *In silico *analysis is hoped to aid in the further understanding of STYKs encoded in various genomes. The diversity of the functional domains occurring in these protein kinase like gene products provide clues to their biological functions that are yet to be explored experimentally. The involvement of bacterial STYKs in various cellular processes described in the previous sections suggests a very little overlap with the Histidine kinases and the protein kinases of the eukaryotes.

Large-scale genomic analysis are fraught with uncertainties related to the inheritance of function among remotely homologous proteins. Nevertheless search procedures employed in the current study for the study of domain organisation of gene products has been extensively bench marked [122] An accuracy level of 96% is achieved by use of stringent search parameters described in 'Materials and Methods' section. The functional roles of STYKs and related systems encoded in various prokaryotic organisms deduced using computational approaches in the present analysis and in a recent independent analysis [123] is hoped to provide useful leads for further studies on their regulation.

Experimental studies [eg., [124]] elucidating the mechanisms of action STYKs and their homologues in various cellular processes as suggested from the analysis would therefore have implications in understanding their influence on the growth and development of the bacteria.

## Methods

The complete set of predicted protein sequences from the ORFs of the bacterial genomes has been obtained from NCBI [125] Using sensitive sequence profile matching algorithms, STYK like sequences have been identified in the genomes.

We have employed multiple sensitive sequence search and analysis methods PSI-BLAST [126], IMPALA [127] and HMMer which matches Hidden Markov Models (HMMs) [128]. These programs have been previously benchmarked [122,129,130] and we have used a stringent cutoff for e-values (0.0005 in PSI_BLAST, 10^-5 ^in IMPALA and 0.01 for HMMer) for identifying homologues of kinases. The list of predicted STYKs used for further analysis has been arrived at after careful cross-referencing between the results of these methods as well as manual scrutiny for a variety of factors such as length of the kinase domains and presence of critical functional residues. Kinase catalytic domains of the PKLA were aligned using CLUSTALW. Manual decisions are taken considering the presence/absence of functional motifs and the lengths of putative kinase domains. Those kinase-like sequences without functional residues, such as an aspartate in the catalytic base position, are segregated separately and are not considered for the detailed analysis. Number of such sequences in various genomes is provided in Table 2.

Domain assignment to the non-catalytic regions of the kinase containing genes has been carried out using the HMM search methods by querying each of the kinase containing sequences against the 6190 protein family HMMs available in the Pfam database [131]. Trans-membrane segments were detected using TMHMM [132].

The 3-dimensional structures were superimposed using STAMP [133].

**Table 1:** Prokaryotes of complete genomic data analysed and the distribution of encoded STYKs and homologues. The list includes ABC1, RI01 and kinases that share significant sequence similarity with STYK family. Number in parenthesis indicates the sequences lacking catalytic Asp and hence are unlikely to function as a kinase.

**Organism**	**STYK**	**ABC1**	**RIO1**
**ARCHAEA**			
*Aeropyrum pernix*	7	-	2
*Archaeoglobus fulgidus*	2	-	2
*Halobacterium salinarium*	5 (1)	1	2
*Methanococcus janaschii*	4	-	2
*Methanopyrus kandleri*	1	-	1
*Methanosarcina acetivorans*	5	2	2
*Methanosarcina mazei Goe1*	4	1	2
*Methanothermobacter thermoautotrophius*	3	1	1
*Nanoarchaeum equitans*	3	-	2
*Pyrobaculum aerophilum*	4	-	2
*Pyrococcus abysii*	4	-	2
*Pyrococcus horikoshii*	4	-	2
*Sulfolobus solfataricus*	7	1	2
*Sulfolobus tokodaii*	11	1	2
*Thermoplasma acidophilum*	3	1	1
*Thermoplasma volcanium*	4	1	2
**BACTERIA**			
*Agrobacterium tumefaciens str. C58*	2	1	-
*Aquifex aeolicus*	2	-	1
*Bacillus anthracis str. Ames*	3	-	-
*Bacillus cereus ATCC 14579*	6	2	-
*Bacillus halodurans*	4	1	-
*Bacillus subtilis*	3	-	-
*Bacteroides thetaiotaomicron VPI-5482*	2	-	-
*Bifidobacterium longum NCC2705*	7	1	-
*Bordetella bronchiseptica*	2	1	-
*Bordetella parapertussis*	2	1	-
*Bordetella pertussis*	2	1	-
*Borrelia burgdorferi*	-	-	-
*Bradyrhizobium japonicum*	5	1	1
*Brucella melitensis*	-	-	-
*Brucella suis*	2	2	-
*Buchnera aphidicola*	-	-	-
*Buchnera sp. APS*	-	-	-
*Candidatus Blochmannia floridanus*	1	1	-
*Caulobacter crescentus*	1	1	-
*Chlamydia muridarium*	1(1)	-	-
*Chlamydophila pneumoniae J138*	3 (1)	-	-
*Chlamydia trachomatis*	3 (1)	-	-
*Chlamydophila caviae GPIC*	3 (1)	-	-
*Chlamydophila pneumoniae AR39*	3 (1)	-	-
*Chlorobium tepidum TLS*	-	-	-
*Chromobacterium violaceum ATCC 12472*	3	2	-
*Clostridium acetobutylicum*	3	1	-
*Clostridium perfringens*	2	1	-
*Clostridium tetani E88*	1	-	-
*Corynebacterium diphtheriae*	4	-	-
*Corynebacterium efficiens YS-314*	4	-	-
*Corynebacterium glutamicum ATCC 13032*	4	-	-
*Coxiella burnetii RSA 493*	4	1	-
*Deinococcus radiodurans*	10	-	1
*Enterococcus faecalis V583*	2	-	-
*Escherichia coli K12*	4	1	-
*Escherichia coli O157:H7 EDL933*	4	1	-
*Fusobacterium nucleatum subsp. nucleatum ATCC 25586*	1	-	-
*Geobacter sulfurreducens PCA*	1	-	-
*Gloeobacter violaceus[B]4.7NC_005125*	19 (1)	4	-
*Haemophilus ducreyi*	2	1	-
*Haemophilus influenzae*	1	-	-
*Helicobacter hepaticus ATCC 51449*	-	-	-
*Helicobacter pylori J99*	1	-	-
*Lactobacillus plantarum WCFS1*	3	1	-
*Lactococcus lactis*	1	-	-
*Leptospira interrogans*	5 (2)	2	-
*Mesorhizobium meliloti*	3	1	-
*Mycobacterium avium subsp. paratuberculosis*	12	2	-
*Mycobacterium bovis*	10	2	-
*Mycobacterium leprae*	6	2	-
*Mycobacterium tuberculosis*	13	2	-
*Mycoplasma gallisepticum*	1	-	-
*Mycoplasma genitalium*	1	-	-
*Mycoplasma penetrans*	1	-	-
*Mycoplasma pneumoniae*	1	-	-
*Mycoplasma pulmonis*	1	-	-
*Neisseria meningititis*	1	1	-
*Nitrosomonas europaea*	2	2	-
*Nostoc sp. PCC 7120*	53	4	-
*Oceanobacillus iheyensis*	1	-	-
*Onion yellows phytoplasma*	-	-	-
*Pasteurella multocida*	2	1	-
*Photorhabdus luminescens subsp. laumondii*	1	1	-
*Pirellula sp.*	59 (1)	2	-
*Porphyromonas gingivalis W83*	1	-	-
*Prochlorococcus marinus MED4*	3	3	-
*Prochlorococcus marinus MIT 9313*	4	3	-
*Prochlorococcus marinus subsp. pastoris str.*	3	3	-
*Pseudomonas aeruginosa*	11	1	1
*Pseudomonas putida KT2440*	14 (2)	-	2
*Pseudomonas syringae*	22	2	2
*Ralstonia solanacearum*	3	1	-
*Rhodopseudomonas palustris*	5 (3)	1	-
*Rickettsia conorii*	1	1	-
*Rickettsia prowazekii*	1	1	-
*Salmonella enterica subsp. enterica serovar Typhi Ty2*	4	1	-
*Salmonella typhimurium LT2*	2	1	-
*Shewanella oneidensis MR-1*	8 (2)	1	1
*Shigella flexneri 2a str. 2457T*	3	1	-
*Sinorhizobium meliloti*	1	1	-
*Staphylococcus aureus*	2	-	-
*Staphylococcus epidermidis ATCC 12228*	1	-	-
*Streptococcus mutans UA159*	1	-	-
*Streptococcus pyogenes M1 GAS*	1	-	-
*Streptococcus pyogenes SSI-1*	1	-	-
*Streptomyces avermitilis*	34 (2)	1	-
*Streptomyces coelicolor A3*	18	-	-
*Synechococcus sp. WH 8102*	3	3	-
*Synechocystis*	12 (1)	5	-
*Thermoanaerobacter tengcongensis*	1	-	-
*Thermosynechococcus elongatus*	14	-	-
*Thermotoga maritima*	1 (1)	1	-
*Treponema pallidum*	-	-	-
*Tropheryma whipplei str Twist*	4 (2)	-	-
*Ureaplasma ureolyticum*	1	-	-
*Vibrio cholerae*	2	1	-
*Vibrio parahaemolyticus RIMD 2210633*	6	1	-
*Vibrio vulnificus CMCP6*	5	1	-
*Wigglesworthia brevipalpis*	1	1	-
*Wolinella succinogenes*	1	1	-
*Xanthomonas axonopodis pv. citri str. 306*	5	-	-
*Xanthomonas campestris pv. campestris str. ATCC 33913*	1	-	-
*Xylella fastidiosa*	3	1	1
*Xylella fastidiosa Temecula1*	3	1	1
*Yersinia pestis*	4	1	1

## Authors' contributions

AK performed the computational sequence analysis and modelling. AK and NS conceived the study and participated in its design and coordination. Both the authors read and approved the final manuscript.

## Supplementary Material

Additional File 1 Data files comprising of the description of protein kinases and homologues encoded in genomes of organisims considered in the current analysis are provided as supplementary information accompanying this article. Each additional data file lists the gene identifiers, length, and domain arrangement of protein kinases and homologues identified in the current analysis. Click here for file
