# Supplementary material for: Diversity in domain architectures of Ser/Thr kinases and their homologues in prokaryotes
Source: BMC Genomics. 2005 Sep 19;6:129. doi: 10.1186/1471-2164-6-129 (PMC1262709; doi:10.1186/1471-2164-6-129)
Supplement: Additional File 1 — Data files comprising of the description of protein kinases and homologues encoded in genomes of organisims considered in the current analysis are provided as supplementary information accompanying this article. Each additional data file lists the gene identifiers, length, and domain arrangement of protein kinases and homologues identified in the current analysis. [file 1471-2164-6-129-S1.tar › Supplementary_files/Bacillus_anthracis_str_ Ames.htm]

Kinases in Bacillus anthracis str. Ames


# Kinases in Bacillus anthracis str. Ames

|  |  |  |  |  |  |  |  |  |  |  |  |  |  |  |  |  |  |  |  |  |  |  |  |
| --- | --- | --- | --- | --- | --- | --- | --- | --- | --- | --- | --- | --- | --- | --- | --- | --- | --- | --- | --- | --- | --- | --- | --- |
| **Gene code** | **Length** | **Domain information** || gi|30258509|gb|AAP27728.1| | 657 | Pkinase     11-267 |
|  |  | TM     i338-360o- |
|  |  | PASTA     367-432 |
|  |  | PASTA     435-499 |
|  |  | PASTA     502-566 |
| gi|30256946|gb|AAP26178.1| | 273 | Pkinase     24-270 |
| gi|30256838|gb|AAP26070.1| | 468 | Pkinase     35-274 |
