# Supplementary material for: Diversity in domain architectures of Ser/Thr kinases and their homologues in prokaryotes
Source: BMC Genomics. 2005 Sep 19;6:129. doi: 10.1186/1471-2164-6-129 (PMC1262709; doi:10.1186/1471-2164-6-129)
Supplement: Additional File 1 — Data files comprising of the description of protein kinases and homologues encoded in genomes of organisims considered in the current analysis are provided as supplementary information accompanying this article. Each additional data file lists the gene identifiers, length, and domain arrangement of protein kinases and homologues identified in the current analysis. [file 1471-2164-6-129-S1.tar › Supplementary_files/Bifidobacterium_longum_NCC2705.htm]

Kinases in Bifidobacterium longum NCC2705


# Kinases in Bifidobacterium longum NCC2705

|  |  |  |  |  |  |  |  |  |  |  |  |  |  |  |  |  |  |  |  |  |  |  |  |  |  |  |  |  |  |  |  |  |  |  |  |  |  |  |  |  |  |  |  |  |  |  |  |  |  |  |  |  |  |  |  |  |  |  |  |
| --- | --- | --- | --- | --- | --- | --- | --- | --- | --- | --- | --- | --- | --- | --- | --- | --- | --- | --- | --- | --- | --- | --- | --- | --- | --- | --- | --- | --- | --- | --- | --- | --- | --- | --- | --- | --- | --- | --- | --- | --- | --- | --- | --- | --- | --- | --- | --- | --- | --- | --- | --- | --- | --- | --- | --- | --- | --- | --- | --- |
| **Gene code** | **Length** | **Domain information** || gi|23325797|gb|AAN24413.1|AE014680\_7 | 690 | Pkinase     14-281 |
|  |  | PASTA     401-467 |
|  |  | PASTA     470-536 |
|  |  | PASTA     537-601 |
|  |  | PASTA     604-665 |
|  |  | TM     i369-391o- |
| gi|23326698|gb|AAN25222.1|AE014772\_9 | 757 | Pkinase     14-276 |
|  |  | PASTA     542-614 |
|  |  | PASTA     618-681 |
|  |  | TM     i426-448o- |
| gi|23325796|gb|AAN24412.1|AE014680\_6 | 316 | Pkinase     13-266 |
| gi|23326452|gb|AAN25003.1|AE014745\_1 | 473 | Pkinase     14-272 |
|  |  | TM     o317-339i- |
| gi|23326147|gb|AAN24730.1|AE014713\_9 | 566 | Pkinase     16-250 |
|  |  | TM     i292-314o- |
| gi|23325873|gb|AAN24482.1|AE014687\_7 | 746 | Pkinase     28-296 |
|  |  | TM     o534-556i604-626o646-668i718-740o- |
| gi|23325629|gb|AAN24264.1|AE014661\_6 | 501 | ABC1     55-174 |
|  |  | TM     o441-460i467-489o- |
