# Supplementary material for: Diversity in domain architectures of Ser/Thr kinases and their homologues in prokaryotes
Source: BMC Genomics. 2005 Sep 19;6:129. doi: 10.1186/1471-2164-6-129 (PMC1262709; doi:10.1186/1471-2164-6-129)
Supplement: Additional File 1 — Data files comprising of the description of protein kinases and homologues encoded in genomes of organisims considered in the current analysis are provided as supplementary information accompanying this article. Each additional data file lists the gene identifiers, length, and domain arrangement of protein kinases and homologues identified in the current analysis. [file 1471-2164-6-129-S1.tar › Supplementary_files/Corynebacterium_efficiens_YS_314.htm]

Kinases in Corynebacterium efficiens YS-314


# Kinases in Corynebacterium efficiens YS-314

|  |  |  |  |  |  |  |  |  |  |  |  |  |  |  |  |  |  |  |  |  |  |  |  |  |  |  |  |  |  |  |  |  |  |  |  |  |  |  |  |  |  |  |  |  |  |  |  |  |  |  |
| --- | --- | --- | --- | --- | --- | --- | --- | --- | --- | --- | --- | --- | --- | --- | --- | --- | --- | --- | --- | --- | --- | --- | --- | --- | --- | --- | --- | --- | --- | --- | --- | --- | --- | --- | --- | --- | --- | --- | --- | --- | --- | --- | --- | --- | --- | --- | --- | --- | --- | --- |
| **Gene code** | **Length** | **Domain information** || gi|23491868|dbj|BAC16843.1| | 660 | Pkinase     9-275 |
|  |  | PASTA     379-443 |
|  |  | PASTA     446-512 |
|  |  | PASTA     515-577 |
|  |  | PASTA     579-650 |
|  |  | TM     i349-371o- |
| gi|23493912|dbj|BAC18880.1| | 741 | Pkinase     14-269 |
|  |  | PASTA     416-480 |
|  |  | PASTA     482-547 |
|  |  | PASTA     550-615 |
|  |  | PASTA     616-682 |
|  |  | PASTA     683-741 |
|  |  | TM     i391-413o- |
| gi|23491869|dbj|BAC16844.1| | 520 | Pkinase     20-281 |
|  |  | TM     i339-361o- |
| gi|23494432|dbj|BAC19399.1| | 848 | Pkinase     201-445 |
