# Supplementary material for: Diversity in domain architectures of Ser/Thr kinases and their homologues in prokaryotes
Source: BMC Genomics. 2005 Sep 19;6:129. doi: 10.1186/1471-2164-6-129 (PMC1262709; doi:10.1186/1471-2164-6-129)
Supplement: Additional File 1 — Data files comprising of the description of protein kinases and homologues encoded in genomes of organisims considered in the current analysis are provided as supplementary information accompanying this article. Each additional data file lists the gene identifiers, length, and domain arrangement of protein kinases and homologues identified in the current analysis. [file 1471-2164-6-129-S1.tar › Supplementary_files/Corynebacterium_glutamicum_ATCC_13032.htm]

Kinases in Corynebacterium glutamicum ATCC 13032


# Kinases in Corynebacterium glutamicum ATCC 13032

|  |  |  |  |  |  |  |  |  |  |  |  |  |  |  |  |  |  |  |  |  |  |  |  |  |  |  |  |  |  |  |  |  |  |  |  |  |  |  |  |  |  |  |  |  |
| --- | --- | --- | --- | --- | --- | --- | --- | --- | --- | --- | --- | --- | --- | --- | --- | --- | --- | --- | --- | --- | --- | --- | --- | --- | --- | --- | --- | --- | --- | --- | --- | --- | --- | --- | --- | --- | --- | --- | --- | --- | --- | --- | --- | --- |
| **Gene code** | **Length** | **Domain information** || gi|19551291|ref|NP\_599293.1| | 646 | Pkinase     9-275 |
|  |  | PASTA     367-431 |
|  |  | PASTA     434-500 |
|  |  | PASTA     503-565 |
| gi|19553377|ref|NP\_601379.1| | 740 | Pkinase     14-272 |
|  |  | PASTA     416-480 |
|  |  | PASTA     482-547 |
|  |  | PASTA     550-615 |
|  |  | PASTA     616-682 |
|  |  | PASTA     683-738 |
|  |  | TM     i391-413o- |
| gi|19551292|ref|NP\_599294.1| | 469 | Pkinase     20-281 |
|  |  | TM     i329-351o- |
| gi|19553944|ref|NP\_601946.1| | 822 | Pkinase     175-419 |
