# Supplementary material for: Diversity in domain architectures of Ser/Thr kinases and their homologues in prokaryotes
Source: BMC Genomics. 2005 Sep 19;6:129. doi: 10.1186/1471-2164-6-129 (PMC1262709; doi:10.1186/1471-2164-6-129)
Supplement: Additional File 1 — Data files comprising of the description of protein kinases and homologues encoded in genomes of organisims considered in the current analysis are provided as supplementary information accompanying this article. Each additional data file lists the gene identifiers, length, and domain arrangement of protein kinases and homologues identified in the current analysis. [file 1471-2164-6-129-S1.tar › Supplementary_files/Deinococcus_radiodurans.htm]

Kinases in Deinococcus radiodurans


# Kinases in Deinococcus radiodurans

|  |  |  |  |  |  |  |  |  |  |  |  |  |  |  |  |  |  |  |  |  |  |  |  |  |  |  |  |  |  |  |  |  |  |  |  |  |  |  |  |  |  |  |  |  |  |  |  |  |  |  |  |  |  |  |  |  |  |  |  |  |  |  |  |  |  |  |  |  |
| --- | --- | --- | --- | --- | --- | --- | --- | --- | --- | --- | --- | --- | --- | --- | --- | --- | --- | --- | --- | --- | --- | --- | --- | --- | --- | --- | --- | --- | --- | --- | --- | --- | --- | --- | --- | --- | --- | --- | --- | --- | --- | --- | --- | --- | --- | --- | --- | --- | --- | --- | --- | --- | --- | --- | --- | --- | --- | --- | --- | --- | --- | --- | --- | --- | --- | --- | --- | --- |
| **Gene code** | **Length** | **Domain information** || gi6457717gbAAF09648.1AE001868\_10 | 591 | Pkinase     11-273 |
|  |  | NHL     384-410 |
|  |  | NHL     430-457 |
|  |  | NHL     478-505 |
| gi6458218gbAAF10106.1AE001911\_1 | 700 | Pkinase     31-268 |
| gi6458957gbAAF10786.1AE001970\_1 | 350 | Pkinase     39-286 |
| gi6458987gbAAF10814.1AE001972\_4 | 278 | Pkinase     18-252 |
| gi6459630gbAAF11404.1AE002025\_3 | 251 | Pkinase     12-242 |
| gi6460339gbAAF12057.1AE002081\_2 | 668 | Pkinase     13-270 |
|  |  | PQQ     369-406 |
|  |  | PQQ     409-446 |
|  |  | PQQ     449-486 |
|  |  | PQQ     561-598 |
|  |  | PQQ     601-638 |
| gi6460012gbAAF11757.1AE002054\_2 | 303 | RIO1     101-287 |
|  |  | Kdo     102-271 |
| gi6460369gbAAF12085.1AE002083\_9 | 679 | Pkinase     30-299 |
|  |  | TM     o392-411i416-438o- |
| gi6460744gbAAF12449.1AE001863\_74 | 524 | Pkinase     50-312 |
|  |  | TM     o418-440i453-475o502-521i- |
| gi6460742gbAAF12447.1AE001863\_72 | 616 | pkinase     155-380 |
|  |  | PP2C     379-607 |
