# Supplementary material for: Diversity in domain architectures of Ser/Thr kinases and their homologues in prokaryotes
Source: BMC Genomics. 2005 Sep 19;6:129. doi: 10.1186/1471-2164-6-129 (PMC1262709; doi:10.1186/1471-2164-6-129)
Supplement: Additional File 1 — Data files comprising of the description of protein kinases and homologues encoded in genomes of organisims considered in the current analysis are provided as supplementary information accompanying this article. Each additional data file lists the gene identifiers, length, and domain arrangement of protein kinases and homologues identified in the current analysis. [file 1471-2164-6-129-S1.tar › Supplementary_files/Gloeobacter_violaceus.htm]

Kinases in Gloeobacter violaceus


# Kinases in Gloeobacter violaceus

|  |  |  |  |  |  |  |  |  |  |  |  |  |  |  |  |  |  |  |  |  |  |  |  |  |  |  |  |  |  |  |  |  |  |  |  |  |  |  |  |  |  |  |  |  |  |  |  |  |  |  |  |  |  |  |  |  |  |  |  |  |  |  |  |  |  |  |  |  |  |  |  |  |  |  |  |  |  |  |  |  |  |  |  |  |  |  |  |  |  |  |  |  |  |  |  |  |  |  |  |  |  |  |  |  |  |  |  |  |  |  |  |  |  |  |  |  |  |  |  |  |  |  |  |  |  |  |  |  |  |  |  |
| --- | --- | --- | --- | --- | --- | --- | --- | --- | --- | --- | --- | --- | --- | --- | --- | --- | --- | --- | --- | --- | --- | --- | --- | --- | --- | --- | --- | --- | --- | --- | --- | --- | --- | --- | --- | --- | --- | --- | --- | --- | --- | --- | --- | --- | --- | --- | --- | --- | --- | --- | --- | --- | --- | --- | --- | --- | --- | --- | --- | --- | --- | --- | --- | --- | --- | --- | --- | --- | --- | --- | --- | --- | --- | --- | --- | --- | --- | --- | --- | --- | --- | --- | --- | --- | --- | --- | --- | --- | --- | --- | --- | --- | --- | --- | --- | --- | --- | --- | --- | --- | --- | --- | --- | --- | --- | --- | --- | --- | --- | --- | --- | --- | --- | --- | --- | --- | --- | --- | --- | --- | --- | --- | --- | --- | --- | --- | --- | --- | --- | --- | --- |
| **Gene code** | **Length** | **Domain information** || gi|37519623|ref|NP\_923000.1| | 340 | Pkinase     19-300 |
|  |  | TM     o321-339i- |
| gi|37520915|ref|NP\_924292.1| | 456 | Pkinase     19-290 |
| gi|37520234|ref|NP\_923611.1| | 386 | Pkinase     18-304 |
| gi|37523641|ref|NP\_927018.1| | 946 | Kdo     76-257 |
|  |  | Pkinase     87-368 |
|  |  | TPR     573-609 |
|  |  | TPR     617-651 |
|  |  | TPR     652-685 |
|  |  | TPR     719-752 |
|  |  | TPR     753-786 |
|  |  | TPR     787-820 |
| gi|37520226|ref|NP\_923603.1| | 456 | Pkinase     19-288 |
| gi|37520484|ref|NP\_923861.1| | 483 | Pkinase     34-284 |
| gi|37521121|ref|NP\_924498.1| | 544 | Pkinase     10-276 |
| gi|37521696|ref|NP\_925073.1| | 280 | Pkinase     15-264 |
| gi|37520154|ref|NP\_923531.1| | 995 | Kdo     75-274 |
|  |  | Pkinase     86-374 |
|  |  | TPR     626-662 |
|  |  | TPR     705-738 |
|  |  | TPR     773-806 |
|  |  | TM     i388-407o417-439i451-470o- |
| gi|37520665|ref|NP\_924042.1| | 913 | Pkinase     81-343 |
|  |  | TPR     538-574 |
|  |  | TPR     616-651 |
|  |  | TPR     720-753 |
|  |  | TPR     754-787 |
|  |  | TPR     797-830 |
| gi|37519991|ref|NP\_923368.1| | 422 | Pkinase     13-262 |
|  |  | TM     o303-325i332-354o- |
| gi|37521672|ref|NP\_925049.1| | 361 | Pkinase     13-262 |
|  |  | TM     o297-319i332-354o- |
| gi|37523676|ref|NP\_927053.1| | 290 | Pkinase     43-284 |
| gi|37523586|ref|NP\_926963.1| | 293 | Pkinase     13-270 |
| gi|37522693|ref|NP\_926070.1| | 616 | ABC1     146-264 |
| gi|37523747|ref|NP\_927124.1| | 595 | ABC1     150-270 |
|  |  | TM     i53-70o536-558i563-585o- |
| gi|37523981|ref|NP\_927358.1| | 562 | ABC1     123-242 |
|  |  | TM     i29-51o- |
| gi|37520000|ref|NP\_923377.1| | 696 | Pkinase     59-316 |
|  |  | TM     i339-361o- |
| gi|37521037|ref|NP\_924414.1| | 545 | ABC1     104-223 |
|  |  | TM     o520-542i- |
