# Supplementary material for: Diversity in domain architectures of Ser/Thr kinases and their homologues in prokaryotes
Source: BMC Genomics. 2005 Sep 19;6:129. doi: 10.1186/1471-2164-6-129 (PMC1262709; doi:10.1186/1471-2164-6-129)
Supplement: Additional File 1 — Data files comprising of the description of protein kinases and homologues encoded in genomes of organisims considered in the current analysis are provided as supplementary information accompanying this article. Each additional data file lists the gene identifiers, length, and domain arrangement of protein kinases and homologues identified in the current analysis. [file 1471-2164-6-129-S1.tar › Supplementary_files/Lactobacillus_plantarum_WCFS1.htm]

Kinases in Lactobacillus plantarum WCFS1


# Kinases in Lactobacillus plantarum WCFS1

|  |  |  |  |  |  |  |  |  |  |  |  |  |  |  |  |  |  |  |  |  |  |  |  |  |  |  |  |  |  |
| --- | --- | --- | --- | --- | --- | --- | --- | --- | --- | --- | --- | --- | --- | --- | --- | --- | --- | --- | --- | --- | --- | --- | --- | --- | --- | --- | --- | --- | --- |
| **Gene code** | **Length** | **Domain information** || gi|28378319|ref|NP\_785211.1| | 674 | Pkinase     11-271 |
|  |  | PASTA     380-445 |
|  |  | PASTA     451-512 |
|  |  | PASTA     515-579 |
|  |  | TM     i356-373o- |
| gi|28379566|ref|NP\_786458.1| | 368 | Pkinase     45-254 |
|  |  | TM     i286-304o319-336i349-366o- |
| gi|28379783|ref|NP\_786675.1| | 578 | ABC1     100-218 |
|  |  | TM     i514-536o551-573i- |
