# Supplementary material for: Diversity in domain architectures of Ser/Thr kinases and their homologues in prokaryotes
Source: BMC Genomics. 2005 Sep 19;6:129. doi: 10.1186/1471-2164-6-129 (PMC1262709; doi:10.1186/1471-2164-6-129)
Supplement: Additional File 1 — Data files comprising of the description of protein kinases and homologues encoded in genomes of organisims considered in the current analysis are provided as supplementary information accompanying this article. Each additional data file lists the gene identifiers, length, and domain arrangement of protein kinases and homologues identified in the current analysis. [file 1471-2164-6-129-S1.tar › Supplementary_files/Leptospira_interrogans_serovar_ai str_56601.htm]

Kinases in Leptospira interrogans serovar lai str. 56601


# Kinases in Leptospira interrogans serovar lai str. 56601

|  |  |  |  |  |  |  |  |  |  |  |  |  |  |  |  |  |  |  |  |  |  |  |  |  |  |  |  |  |  |  |  |  |  |  |  |  |  |  |  |  |  |  |  |  |  |  |  |
| --- | --- | --- | --- | --- | --- | --- | --- | --- | --- | --- | --- | --- | --- | --- | --- | --- | --- | --- | --- | --- | --- | --- | --- | --- | --- | --- | --- | --- | --- | --- | --- | --- | --- | --- | --- | --- | --- | --- | --- | --- | --- | --- | --- | --- | --- | --- | --- |
| **Gene code** | **Length** | **Domain information** || gi|24195011|gb|AAN48621.1|AE011322\_4 | 1780 | Pkinase     6-273 |
|  |  | KAP\_NTPase     301-597 |
|  |  | TPR     809-842 |
|  |  | TPR     961-994 |
|  |  | GAF     1355-1498 |
|  |  | SpoIIE     1580-1780 |
| gi|24194708|gb|AAN48363.1|AE011299\_4 | 1759 | Pkinase     9-268 |
|  |  | GAF     1345-1489 |
|  |  | SpoIIE     1570-1759 |
| gi|24197004|gb|AAN50311.1|AE011474\_1 | 1731 | Pkinase     13-262 |
|  |  | GAF     1324-1465 |
|  |  | SpoIIE     1540-1731 |
| gi|24196371|gb|AAN49776.1|AE011425\_1 | 442 | ABC1     113-234 |
| gi|24194906|gb|AAN48534.1|AE011313\_10 | 570 | ABC1     123-238 |
|  |  | TM     o419-441i509-528o538-559i- |
