# Supplementary material for: Diversity in domain architectures of Ser/Thr kinases and their homologues in prokaryotes
Source: BMC Genomics. 2005 Sep 19;6:129. doi: 10.1186/1471-2164-6-129 (PMC1262709; doi:10.1186/1471-2164-6-129)
Supplement: Additional File 1 — Data files comprising of the description of protein kinases and homologues encoded in genomes of organisims considered in the current analysis are provided as supplementary information accompanying this article. Each additional data file lists the gene identifiers, length, and domain arrangement of protein kinases and homologues identified in the current analysis. [file 1471-2164-6-129-S1.tar › Supplementary_files/Methanosarcina_acetivorans_str_ C2A.htm]

Kinases in Methanosarcina acetivorans str. C2A


# Kinases in Methanosarcina acetivorans str. C2A

|  |  |  |  |  |  |  |  |  |  |  |  |  |  |  |  |  |  |  |  |  |  |  |  |  |  |  |  |  |  |
| --- | --- | --- | --- | --- | --- | --- | --- | --- | --- | --- | --- | --- | --- | --- | --- | --- | --- | --- | --- | --- | --- | --- | --- | --- | --- | --- | --- | --- | --- |
| **Gene code** | **Length** | **Domain information** || gi|19915764|gb|AAM05266.1| | 559 | ABC1     117-236 |
|  |  | TM     o529-551i- |
| gi|19914718|gb|AAM04333.1| | 262 | RIO1     67-252 |
| gi|19915174|gb|AAM04741.1| | 551 | ABC1     132-251 |
|  |  | TM     i504-523o527-549i- |
| gi|19917773|gb|AAM07060.1| | 547 | Peptidase\_M22     39-122 |
|  |  | Kdo     353-519 |
|  |  | RIO1     362-501 |
| gi|19916478|gb|AAM05901.1| | 307 | RIO1     107-289 |
