# Supplementary material for: Diversity in domain architectures of Ser/Thr kinases and their homologues in prokaryotes
Source: BMC Genomics. 2005 Sep 19;6:129. doi: 10.1186/1471-2164-6-129 (PMC1262709; doi:10.1186/1471-2164-6-129)
Supplement: Additional File 1 — Data files comprising of the description of protein kinases and homologues encoded in genomes of organisims considered in the current analysis are provided as supplementary information accompanying this article. Each additional data file lists the gene identifiers, length, and domain arrangement of protein kinases and homologues identified in the current analysis. [file 1471-2164-6-129-S1.tar › Supplementary_files/Mycobacterium_avium_subsp_ paratuberculosis_str_ k10.htm]

Kinases in Mycobacterium avium subsp. paratuberculosis str. k10


# Kinases in Mycobacterium avium subsp. paratuberculosis str. k10

|  |  |  |  |  |  |  |  |  |  |  |  |  |  |  |  |  |  |  |  |  |  |  |  |  |  |  |  |  |  |  |  |  |  |  |  |  |  |  |  |  |  |  |  |  |  |  |  |  |  |  |  |  |  |  |  |  |  |  |  |  |  |  |  |  |  |  |  |  |  |  |  |  |  |  |  |  |  |  |  |  |  |  |  |  |  |  |  |  |  |  |  |  |
| --- | --- | --- | --- | --- | --- | --- | --- | --- | --- | --- | --- | --- | --- | --- | --- | --- | --- | --- | --- | --- | --- | --- | --- | --- | --- | --- | --- | --- | --- | --- | --- | --- | --- | --- | --- | --- | --- | --- | --- | --- | --- | --- | --- | --- | --- | --- | --- | --- | --- | --- | --- | --- | --- | --- | --- | --- | --- | --- | --- | --- | --- | --- | --- | --- | --- | --- | --- | --- | --- | --- | --- | --- | --- | --- | --- | --- | --- | --- | --- | --- | --- | --- | --- | --- | --- | --- | --- | --- | --- | --- | --- | --- |
| **Gene code** | **Length** | **Domain information** || gi|41406114|ref|NP\_958950.1| | 626 | Pkinase     11-273 |
|  |  | PASTA     358-422 |
|  |  | PASTA     425-490 |
|  |  | PASTA     493-557 |
|  |  | PASTA     559-626 |
|  |  | TM     i332-354o- |
| gi|41408012|ref|NP\_960848.1| | 396 | Pkinase     6-262 |
|  |  | TM     i367-389o- |
| gi|41407147|ref|NP\_959983.1| | 550 | Pkinase     11-272 |
|  |  | Pro\_isomerase     384-550 |
|  |  | TM     i295-317o- |
| gi|41408124|ref|NP\_960960.1| | 514 | Pkinase     12-271 |
|  |  | TM     o325-347i- |
| gi|41408602|ref|NP\_961438.1| | 401 | Kdo     16-205 |
|  |  | Pkinase     16-276 |
| gi|41409485|ref|NP\_962321.1| | 656 | Kdo     10-200 |
|  |  | Pkinase     17-278 |
|  |  | NHL     417-445 |
|  |  | NHL     460-486 |
|  |  | NHL     501-528 |
|  |  | NHL     543-570 |
|  |  | NHL     585-612 |
|  |  | NHL     627-654 |
|  |  | TM     i370-392o- |
| gi|41407430|ref|NP\_960266.1| | 620 | Pkinase     12-276 |
| gi|41406116|ref|NP\_958952.1| | 253 | Pkinase     13-253 |
| gi|41408129|ref|NP\_960965.1| | 485 | Pkinase     17-268 |
| gi|41409991|ref|NP\_962827.1| | 763 | Pkinase     164-406 |
| gi|41410217|ref|NP\_963053.1| | 452 | ABC1     110-231 |
| gi|41409393|ref|NP\_962229.1| | 447 | ABC1     117-233 |
