# Supplementary material for: Diversity in domain architectures of Ser/Thr kinases and their homologues in prokaryotes
Source: BMC Genomics. 2005 Sep 19;6:129. doi: 10.1186/1471-2164-6-129 (PMC1262709; doi:10.1186/1471-2164-6-129)
Supplement: Additional File 1 — Data files comprising of the description of protein kinases and homologues encoded in genomes of organisims considered in the current analysis are provided as supplementary information accompanying this article. Each additional data file lists the gene identifiers, length, and domain arrangement of protein kinases and homologues identified in the current analysis. [file 1471-2164-6-129-S1.tar › Supplementary_files/Mycobacterium_bovis_subsp_bovi_ AF2.htm]

Kinases in Mycobacterium bovis subsp. bovis AF2


# Kinases in Mycobacterium bovis subsp. bovis AF2

|  |  |  |  |  |  |  |  |  |  |  |  |  |  |  |  |  |  |  |  |  |  |  |  |  |  |  |  |  |  |  |  |  |  |  |  |  |  |  |  |  |  |  |  |  |  |  |  |  |  |  |  |  |  |  |  |  |  |  |  |  |  |  |  |  |  |  |  |  |  |  |  |
| --- | --- | --- | --- | --- | --- | --- | --- | --- | --- | --- | --- | --- | --- | --- | --- | --- | --- | --- | --- | --- | --- | --- | --- | --- | --- | --- | --- | --- | --- | --- | --- | --- | --- | --- | --- | --- | --- | --- | --- | --- | --- | --- | --- | --- | --- | --- | --- | --- | --- | --- | --- | --- | --- | --- | --- | --- | --- | --- | --- | --- | --- | --- | --- | --- | --- | --- | --- | --- | --- | --- | --- |
| **Gene code** | **Length** | **Domain information** || gi|31791191|ref|NP\_853684.1| | 626 | Pkinase     11-273 |
|  |  | PASTA     358-422 |
|  |  | PASTA     425-490 |
|  |  | PASTA     493-557 |
|  |  | PASTA     559-626 |
|  |  | TM     i332-354o- |
| gi|31792458|ref|NP\_854951.1| | 596 | Kdo     16-205 |
|  |  | Pkinase     16-276 |
|  |  | TM     i372-394o- |
| gi|31793354|ref|NP\_855847.1| | 399 | Pkinase     19-275 |
|  |  | TM     i370-392o- |
| gi|31791192|ref|NP\_853685.1| | 431 | Pkinase     13-274 |
|  |  | TM     o338-360i- |
| gi|31792931|ref|NP\_855424.1| | 566 | Kdo     6-205 |
|  |  | Pkinase     16-292 |
|  |  | DSBA     400-566 |
|  |  | TM     o337-359i- |
| gi|31792934|ref|NP\_855427.1| | 476 | Pkinase     12-279 |
|  |  | TM     o307-326i- |
| gi|31793271|ref|NP\_855764.1| | 589 | Pkinase     14-279 |
| gi|31791588|ref|NP\_854081.1| | 750 | Pkinase     151-392 |
| gi|31791831|ref|NP\_854324.1| | 488 | ABC1     150-271 |
| gi|31794372|ref|NP\_856865.1| | 447 | ABC1     117-233 |
