# Supplementary material for: Diversity in domain architectures of Ser/Thr kinases and their homologues in prokaryotes
Source: BMC Genomics. 2005 Sep 19;6:129. doi: 10.1186/1471-2164-6-129 (PMC1262709; doi:10.1186/1471-2164-6-129)
Supplement: Additional File 1 — Data files comprising of the description of protein kinases and homologues encoded in genomes of organisims considered in the current analysis are provided as supplementary information accompanying this article. Each additional data file lists the gene identifiers, length, and domain arrangement of protein kinases and homologues identified in the current analysis. [file 1471-2164-6-129-S1.tar › Supplementary_files/Mycobacterium_tuberculosis.htm]

Kinases in Mycobacterium tuberculosis


# Kinases in Mycobacterium tuberculosis

|  |  |  |  |  |  |  |  |  |  |  |  |  |  |  |  |  |  |  |  |  |  |  |  |  |  |  |  |  |  |  |  |  |  |  |  |  |  |  |  |  |  |  |  |  |  |  |  |  |  |  |  |  |  |  |  |  |  |  |  |  |  |  |  |  |  |  |  |  |  |  |  |  |  |  |  |  |  |  |  |  |  |  |  |  |  |  |  |  |  |  |  |  |  |  |  |  |  |  |  |  |  |  |  |  |
| --- | --- | --- | --- | --- | --- | --- | --- | --- | --- | --- | --- | --- | --- | --- | --- | --- | --- | --- | --- | --- | --- | --- | --- | --- | --- | --- | --- | --- | --- | --- | --- | --- | --- | --- | --- | --- | --- | --- | --- | --- | --- | --- | --- | --- | --- | --- | --- | --- | --- | --- | --- | --- | --- | --- | --- | --- | --- | --- | --- | --- | --- | --- | --- | --- | --- | --- | --- | --- | --- | --- | --- | --- | --- | --- | --- | --- | --- | --- | --- | --- | --- | --- | --- | --- | --- | --- | --- | --- | --- | --- | --- | --- | --- | --- | --- | --- | --- | --- | --- | --- | --- | --- | --- | --- |
| **Gene code** | **Length** | **Domain information** || gi1370255gnlPIDe247094 | 589 | Pkinase     14-279 |
| gi1552569gnlPIDe266933 | 626 | Pkinase     11-273 |
|  |  | PASTA     358-422 |
|  |  | PASTA     425-490 |
|  |  | PASTA     493-557 |
|  |  | PASTA     559-626 |
|  |  | TM     i332-354o- |
| gi1552570gnlPIDe267386 | 431 | Pkinase     13-274 |
|  |  | TM     o338-360i- |
| gi1817676gnlPIDe301456 | 750 | Pkinase     151-392 |
| gi2078052gnlPIDe315211 | 664 | Pkinase     15-276 |
|  |  | NHL     425-453 |
|  |  | NHL     468-494 |
|  |  | NHL     509-536 |
|  |  | NHL     551-578 |
|  |  | NHL     593-620 |
|  |  | NHL     635-662 |
|  |  | TM     o380-402i- |
| gi2131007gnlPIDe318983 | 566 | Kdo     6-205 |
|  |  | Pkinase     16-292 |
|  |  | DSBA     400-566 |
|  |  | TM     o337-359i- |
| gi2131011gnlPIDe318987 | 476 | Pkinase     12-279 |
|  |  | TM     o307-326i- |
| gi2911096gnlPIDe1253249 | 399 | Pkinase     19-275 |
|  |  | TM     i370-392o- |
| gi3261596gnlPIDe1299883 | 626 | Kdo     16-205 |
|  |  | Pkinase     16-276 |
|  |  | TM     i404-426o- |
| gi3261603gnlPIDe1299928 | 585 | Pkinase     12-251 |
|  |  | TM     o350-372i- |
| gi3261694gnlPIDe1299858 | 1110 | Pkinase     26-282 |
| gi2827607gnlPIDe1248788 | 447 | ABC1     117-233 |
| gi3261724gnlPIDe1299803 | 488 | ABC1     150-271 |
