# Supplementary material for: Diversity in domain architectures of Ser/Thr kinases and their homologues in prokaryotes
Source: BMC Genomics. 2005 Sep 19;6:129. doi: 10.1186/1471-2164-6-129 (PMC1262709; doi:10.1186/1471-2164-6-129)
Supplement: Additional File 1 — Data files comprising of the description of protein kinases and homologues encoded in genomes of organisims considered in the current analysis are provided as supplementary information accompanying this article. Each additional data file lists the gene identifiers, length, and domain arrangement of protein kinases and homologues identified in the current analysis. [file 1471-2164-6-129-S1.tar › Supplementary_files/Nostoc_sp_PCC_7120.htm]

Kinases in Nostoc sp. PCC 7120


# Kinases in Nostoc sp. PCC 7120

|  |  |  |  |  |  |  |  |  |  |  |  |  |  |  |  |  |  |  |  |  |  |  |  |  |  |  |  |  |  |  |  |  |  |  |  |  |  |  |  |  |  |  |  |  |  |  |  |  |  |  |  |  |  |  |  |  |  |  |  |  |  |  |  |  |  |  |  |  |  |  |  |  |  |  |  |  |  |  |  |  |  |  |  |  |  |  |  |  |  |  |  |  |  |  |  |  |  |  |  |  |  |  |  |  |  |  |  |  |  |  |  |  |  |  |  |  |  |  |  |  |  |  |  |  |  |  |  |  |  |  |  |  |  |  |  |  |  |  |  |  |  |  |  |  |  |  |  |  |  |  |  |  |  |  |  |  |  |  |  |  |  |  |  |  |  |  |  |  |  |  |  |  |  |  |  |  |  |  |  |  |  |  |  |  |  |  |  |  |  |  |  |  |  |  |  |  |  |  |  |  |  |  |  |  |  |  |  |  |  |  |  |  |  |  |  |  |  |  |  |  |  |  |  |  |  |  |  |  |  |  |  |  |  |  |  |  |  |  |  |  |  |  |  |  |  |  |  |  |  |  |  |  |  |  |  |  |  |  |  |  |  |  |  |  |  |  |  |  |  |  |  |  |  |  |  |  |  |  |  |  |  |  |  |  |  |  |  |  |  |  |  |  |  |  |  |  |  |  |  |  |  |  |  |  |  |  |  |  |  |  |  |  |  |  |  |  |  |  |  |  |  |  |  |  |  |  |  |  |  |  |  |  |  |  |  |  |  |  |  |  |  |  |  |  |  |  |  |  |  |  |  |  |  |  |  |  |  |  |  |  |  |  |  |  |  |  |  |  |  |  |  |  |  |  |  |  |  |  |  |  |  |  |  |  |  |  |  |  |  |  |  |  |  |  |  |  |  |  |  |  |  |  |  |  |  |  |  |  |  |  |  |  |  |  |  |  |  |  |  |  |  |  |  |  |  |  |  |  |  |  |  |  |  |  |  |  |  |  |  |  |  |  |  |  |  |  |  |  |  |  |  |  |  |  |  |  |  |  |  |  |  |  |  |  |  |  |  |  |  |  |  |  |  |  |  |  |  |  |  |
| --- | --- | --- | --- | --- | --- | --- | --- | --- | --- | --- | --- | --- | --- | --- | --- | --- | --- | --- | --- | --- | --- | --- | --- | --- | --- | --- | --- | --- | --- | --- | --- | --- | --- | --- | --- | --- | --- | --- | --- | --- | --- | --- | --- | --- | --- | --- | --- | --- | --- | --- | --- | --- | --- | --- | --- | --- | --- | --- | --- | --- | --- | --- | --- | --- | --- | --- | --- | --- | --- | --- | --- | --- | --- | --- | --- | --- | --- | --- | --- | --- | --- | --- | --- | --- | --- | --- | --- | --- | --- | --- | --- | --- | --- | --- | --- | --- | --- | --- | --- | --- | --- | --- | --- | --- | --- | --- | --- | --- | --- | --- | --- | --- | --- | --- | --- | --- | --- | --- | --- | --- | --- | --- | --- | --- | --- | --- | --- | --- | --- | --- | --- | --- | --- | --- | --- | --- | --- | --- | --- | --- | --- | --- | --- | --- | --- | --- | --- | --- | --- | --- | --- | --- | --- | --- | --- | --- | --- | --- | --- | --- | --- | --- | --- | --- | --- | --- | --- | --- | --- | --- | --- | --- | --- | --- | --- | --- | --- | --- | --- | --- | --- | --- | --- | --- | --- | --- | --- | --- | --- | --- | --- | --- | --- | --- | --- | --- | --- | --- | --- | --- | --- | --- | --- | --- | --- | --- | --- | --- | --- | --- | --- | --- | --- | --- | --- | --- | --- | --- | --- | --- | --- | --- | --- | --- | --- | --- | --- | --- | --- | --- | --- | --- | --- | --- | --- | --- | --- | --- | --- | --- | --- | --- | --- | --- | --- | --- | --- | --- | --- | --- | --- | --- | --- | --- | --- | --- | --- | --- | --- | --- | --- | --- | --- | --- | --- | --- | --- | --- | --- | --- | --- | --- | --- | --- | --- | --- | --- | --- | --- | --- | --- | --- | --- | --- | --- | --- | --- | --- | --- | --- | --- | --- | --- | --- | --- | --- | --- | --- | --- | --- | --- | --- | --- | --- | --- | --- | --- | --- | --- | --- | --- | --- | --- | --- | --- | --- | --- | --- | --- | --- | --- | --- | --- | --- | --- | --- | --- | --- | --- | --- | --- | --- | --- | --- | --- | --- | --- | --- | --- | --- | --- | --- | --- | --- | --- | --- | --- | --- | --- | --- | --- | --- | --- | --- | --- | --- | --- | --- | --- | --- | --- | --- | --- | --- | --- | --- | --- | --- | --- | --- | --- | --- | --- | --- | --- | --- | --- | --- | --- | --- | --- | --- | --- | --- | --- | --- | --- | --- | --- | --- | --- | --- | --- | --- | --- | --- | --- | --- | --- | --- | --- | --- | --- | --- | --- | --- | --- | --- | --- | --- | --- | --- | --- | --- | --- | --- | --- | --- | --- | --- | --- | --- | --- | --- | --- | --- | --- | --- | --- | --- | --- | --- | --- | --- | --- | --- | --- | --- | --- | --- | --- | --- | --- | --- | --- | --- | --- | --- | --- | --- | --- | --- | --- | --- | --- | --- | --- | --- | --- | --- | --- | --- | --- | --- | --- | --- | --- | --- | --- | --- | --- | --- | --- | --- | --- | --- | --- | --- | --- |
| **Gene code** | **Length** | **Domain information** || gi|17133502|dbj|BAB76065.1| | 564 | Pkinase     9-270 |
|  |  | TM     o333-355i- |
| gi|17132302|dbj|BAB74906.1| | 534 | Pkinase     20-296 |
| gi|17132301|dbj|BAB74905.1| | 508 | Pkinase     19-294 |
| gi|17130055|dbj|BAB72667.1| | 1799 | Pkinase     12-272 |
|  |  | GAF     1356-1498 |
|  |  | HisKA     1536-1633 |
|  |  | HATPase\_c     1678-1799 |
| gi|17129699|dbj|BAB72312.1| | 1804 | Pkinase     7-274 |
|  |  | GAF     1356-1505 |
|  |  | HATPase\_c     1678-1799 |
| gi|17133132|dbj|BAB75696.1| | 496 | Pkinase     10-271 |
|  |  | TM     i314-336o- |
| gi|17130054|dbj|BAB72666.1| | 1796 | Pkinase     12-272 |
|  |  | GAF     1353-1495 |
|  |  | HisKA     1533-1600 |
|  |  | HATPase\_c     1675-1796 |
| gi|17133950|dbj|BAB76512.1| | 642 | Pkinase     10-269 |
| gi|17131349|dbj|BAB73957.1| | 1777 | Pkinase     7-266 |
|  |  | GAF     1319-1462 |
|  |  | HisKA     1511-1608 |
|  |  | HATPase\_c     1653-1775 |
| gi|17132690|dbj|BAB75256.1| | 1804 | Pkinase     14-272 |
|  |  | GAF     1359-1508 |
|  |  | HisKA     1539-1636 |
|  |  | HATPase\_c     1681-1802 |
| gi|17130246|dbj|BAB72857.1| | 1800 | Pkinase     19-287 |
|  |  | GAF     1366-1506 |
|  |  | HisKA     1537-1634 |
|  |  | HATPase\_c     1679-1800 |
| gi|17131374|dbj|BAB73981.1| | 1783 | Pkinase     7-264 |
|  |  | GAF     1335-1476 |
|  |  | HisKA     1518-1607 |
|  |  | HATPase\_c     1654-1771 |
| gi|17133654|dbj|BAB76217.1| | 520 | Pkinase     14-289 |
| gi|17129668|dbj|BAB72281.1| | 1808 | Pkinase     12-290 |
|  |  | GAF     1356-1498 |
|  |  | HisKA     1547-1644 |
|  |  | HATPase\_c     1683-1806 |
| gi|17130231|dbj|BAB72843.1| | 1850 | Pkinase     7-267 |
|  |  | GAF     1364-1508 |
|  |  | HisKA     1564-1661 |
|  |  | HATPase\_c     1708-1827 |
| gi|17132825|dbj|BAB75390.1| | 2021 | Pkinase     19-307 |
|  |  | GAF     1382-1531 |
|  |  | GAF     1569-1713 |
|  |  | HATPase\_c     1893-2014 |
| gi|17131775|dbj|BAB74381.1| | 1946 | Pkinase     7-276 |
|  |  | GAF     1366-1510 |
|  |  | PAS     1524-1591 |
|  |  | HATPase\_c     1826-1946 |
| gi|17131009|dbj|BAB73618.1| | 546 | Pkinase     12-266 |
| gi|17134417|dbj|BAB76977.1| | 376 | Pkinase     34-289 |
| gi|17133805|dbj|BAB76367.1| | 560 | Pkinase     15-275 |
| gi|17133504|dbj|BAB76067.1| | 597 | Pkinase     10-271 |
| gi|17131426|dbj|BAB74033.1| | 484 | Pkinase     10-268 |
|  |  | TM     o342-364i371-393o406-423i428-445o455-477i- |
| gi|17131504|dbj|BAB74111.1| | 704 | Pkinase     45-316 |
| gi|17133824|dbj|BAB76386.1| | 1900 | Pkinase     14-277 |
|  |  | GAF     1419-1566 |
|  |  | HisKA     1629-1726 |
|  |  | HATPase\_c     1771-1900 |
| gi|17132363|dbj|BAB74967.1| | 524 | Pkinase     34-295 |
|  |  | Pentapeptide     418-457 |
|  |  | Pentapeptide     458-497 |
| gi|17131503|dbj|BAB74110.1| | 535 | Pkinase     9-275 |
| gi|17131350|dbj|BAB73958.1| | 461 | Pkinase     10-269 |
| gi|17135445|dbj|BAB77991.1| | 1787 | Pkinase     13-270 |
|  |  | GAF     1354-1494 |
|  |  | HisKA     1525-1622 |
|  |  | HATPase\_c     1667-1787 |
| gi|17132214|dbj|BAB74818.1| | 676 | Pkinase     36-299 |
|  |  | WD40     382-419 |
|  |  | WD40     424-461 |
|  |  | WD40     466-503 |
|  |  | WD40     508-545 |
|  |  | WD40     550-587 |
|  |  | WD40     592-630 |
|  |  | WD40     635-673 |
| gi|17129783|dbj|BAB72396.1| | 677 | Pkinase     36-293 |
|  |  | WD40     385-422 |
|  |  | WD40     427-464 |
|  |  | WD40     469-506 |
|  |  | WD40     511-548 |
|  |  | WD40     553-590 |
|  |  | WD40     595-632 |
|  |  | WD40     637-674 |
| gi|17133276|dbj|BAB75840.1| | 370 | Pkinase     14-267 |
| gi|17129893|dbj|BAB72506.1| | 427 | FHA     26-92 |
|  |  | Pkinase     171-423 |
| gi|17131594|dbj|BAB74201.1| | 682 | Kdo     27-206 |
|  |  | Pkinase     34-317 |
|  |  | Pectinesterase     367-512 |
| gi|17130658|dbj|BAB73268.1| | 374 | Pkinase     49-309 |
| gi|17133011|dbj|BAB75576.1| | 463 | Pkinase     10-265 |
|  |  | TM     o309-331i338-360o- |
| gi|17134092|dbj|BAB76653.1| | 456 | FHA     27-111 |
|  |  | Pkinase     172-450 |
| gi|17133975|dbj|BAB76537.1| | 777 | CHASE2     63-355 |
|  |  | Pkinase     483-741 |
|  |  | TM     i42-64o352-374i386-408o- |
| gi|17135170|dbj|BAB77716.1| | 553 | Pkinase     36-285 |
|  |  | TM     i324-346o- |
| gi|17132840|dbj|BAB75405.1| | 546 | Pkinase     8-278 |
|  |  | TM     i338-360o390-412i419-441o451-468i473-495o515-537i- |
| gi|17134087|dbj|BAB76648.1| | 452 | Pkinase     16-271 |
|  |  | GUN4     294-435 |
| gi|17130959|dbj|BAB73568.1| | 746 | CHASE2     50-331 |
|  |  | Pkinase     475-733 |
|  |  | TM     i24-46o331-353i360-382o387-409i- |
| gi|17132907|dbj|BAB75472.1| | 707 | Pkinase     10-268 |
|  |  | TPR     329-362 |
|  |  | NSF     348-359 |
|  |  | TPR     363-396 |
|  |  | NSF     382-393 |
|  |  | TPR     397-430 |
|  |  | TPR     431-464 |
|  |  | NSF     450-461 |
|  |  | TPR     465-498 |
|  |  | NSF     484-495 |
|  |  | TPR     499-532 |
|  |  | TPR     533-566 |
|  |  | TPR     567-600 |
|  |  | NSF     586-597 |
|  |  | TPR     601-634 |
|  |  | NSF     620-631 |
|  |  | TPR     635-668 |
|  |  | TM     i299-321o- |
| gi|17130683|dbj|BAB73293.1| | 616 | Pkinase     37-303 |
|  |  | DUF323     375-613 |
| gi|17129689|dbj|BAB72302.1| | 796 | Pkinase     56-304 |
|  |  | ANF\_receptor     441-777 |
| gi|17132866|dbj|BAB75431.1| | 567 | Pkinase     10-272 |
| gi|17132264|dbj|BAB74868.1| | 559 | Pkinase     3-245 |
|  |  | WD40     262-297 |
|  |  | WD40     302-339 |
|  |  | WD40     344-381 |
|  |  | WD40     386-423 |
|  |  | WD40     432-469 |
|  |  | WD40     474-511 |
|  |  | WD40     516-553 |
| gi|17131853|dbj|BAB74459.1| | 334 | Pkinase     24-274 |
|  |  | TM     o297-319i- |
| gi|17135770|dbj|BAB78316.1| | 584 | Pkinase     21-279 |
|  |  | TPR     321-354 |
|  |  | TPR     425-458 |
|  |  | TPR     496-529 |
| gi|17129937|dbj|BAB72550.1| | 572 | ABC1     120-239 |
| gi|17134098|dbj|BAB76659.1| | 670 | ABC1     165-283 |
| gi|17133651|dbj|BAB76214.1| | 589 | ABC1     148-267 |
|  |  | TM     i534-553o558-580i- |
| gi|17135167|dbj|BAB77713.1| | 547 | ABC1     99-217 |
|  |  | TM     i493-510o520-542i- |
| gi|17131918|dbj|BAB74523.1| | 732 | Pkinase     13-303 |
|  |  | TPR     454-487 |
|  |  | TPR     488-521 |
|  |  | TPR     522-563 |
|  |  | TPR     564-605 |
|  |  | TPR     606-647 |
