# Supplementary material for: Diversity in domain architectures of Ser/Thr kinases and their homologues in prokaryotes
Source: BMC Genomics. 2005 Sep 19;6:129. doi: 10.1186/1471-2164-6-129 (PMC1262709; doi:10.1186/1471-2164-6-129)
Supplement: Additional File 1 — Data files comprising of the description of protein kinases and homologues encoded in genomes of organisims considered in the current analysis are provided as supplementary information accompanying this article. Each additional data file lists the gene identifiers, length, and domain arrangement of protein kinases and homologues identified in the current analysis. [file 1471-2164-6-129-S1.tar › Supplementary_files/Pirellula_sp_1.htm]

Kinases in Pirellula sp. 1


# Kinases in Pirellula sp. 1

|  |  |  |  |  |  |  |  |  |  |  |  |  |  |  |  |  |  |  |  |  |  |  |  |  |  |  |  |  |  |  |  |  |  |  |  |  |  |  |  |  |  |  |  |  |  |  |  |  |  |  |  |  |  |  |  |  |  |  |  |  |  |  |  |  |  |  |  |  |  |  |  |  |  |  |  |  |  |  |  |  |  |  |  |  |  |  |  |  |  |  |  |  |  |  |  |  |  |  |  |  |  |  |  |  |  |  |  |  |  |  |  |  |  |  |  |  |  |  |  |  |  |  |  |  |  |  |  |  |  |  |  |  |  |  |  |  |  |  |  |  |  |  |  |  |  |  |  |  |  |  |  |  |  |  |  |  |  |  |  |  |  |  |  |  |  |  |  |  |  |  |  |  |  |  |  |  |  |  |  |  |  |  |  |  |  |  |  |  |  |  |  |  |  |  |  |  |  |  |  |  |  |  |  |  |  |  |  |  |  |  |  |  |  |  |  |  |  |  |  |  |  |  |  |  |  |  |  |  |  |  |  |  |  |  |  |  |  |  |  |  |  |  |  |  |  |  |  |  |  |  |  |  |  |  |  |  |  |  |  |  |  |  |  |  |  |  |  |  |  |  |  |  |  |  |  |  |  |  |  |  |  |  |  |  |  |  |  |  |  |  |  |  |  |  |  |  |  |  |  |  |  |  |  |  |  |  |  |  |  |  |  |  |  |  |  |  |  |  |  |  |  |  |  |  |  |  |  |  |  |  |  |  |  |  |  |  |  |  |  |  |  |  |  |  |  |  |  |  |  |  |  |  |  |  |  |  |  |  |  |  |  |  |  |  |  |  |  |  |  |  |  |  |  |  |  |  |  |  |  |  |  |  |  |  |  |  |  |  |  |  |  |  |  |  |  |  |  |  |  |  |  |  |  |  |  |  |  |  |  |  |  |  |  |  |  |  |  |  |  |  |  |  |  |  |  |  |  |  |  |  |  |  |  |  |  |  |  |  |  |  |  |  |  |  |  |  |  |  |  |  |  |  |  |  |  |  |  |  |  |  |  |  |  |  |  |  |  |  |  |  |
| --- | --- | --- | --- | --- | --- | --- | --- | --- | --- | --- | --- | --- | --- | --- | --- | --- | --- | --- | --- | --- | --- | --- | --- | --- | --- | --- | --- | --- | --- | --- | --- | --- | --- | --- | --- | --- | --- | --- | --- | --- | --- | --- | --- | --- | --- | --- | --- | --- | --- | --- | --- | --- | --- | --- | --- | --- | --- | --- | --- | --- | --- | --- | --- | --- | --- | --- | --- | --- | --- | --- | --- | --- | --- | --- | --- | --- | --- | --- | --- | --- | --- | --- | --- | --- | --- | --- | --- | --- | --- | --- | --- | --- | --- | --- | --- | --- | --- | --- | --- | --- | --- | --- | --- | --- | --- | --- | --- | --- | --- | --- | --- | --- | --- | --- | --- | --- | --- | --- | --- | --- | --- | --- | --- | --- | --- | --- | --- | --- | --- | --- | --- | --- | --- | --- | --- | --- | --- | --- | --- | --- | --- | --- | --- | --- | --- | --- | --- | --- | --- | --- | --- | --- | --- | --- | --- | --- | --- | --- | --- | --- | --- | --- | --- | --- | --- | --- | --- | --- | --- | --- | --- | --- | --- | --- | --- | --- | --- | --- | --- | --- | --- | --- | --- | --- | --- | --- | --- | --- | --- | --- | --- | --- | --- | --- | --- | --- | --- | --- | --- | --- | --- | --- | --- | --- | --- | --- | --- | --- | --- | --- | --- | --- | --- | --- | --- | --- | --- | --- | --- | --- | --- | --- | --- | --- | --- | --- | --- | --- | --- | --- | --- | --- | --- | --- | --- | --- | --- | --- | --- | --- | --- | --- | --- | --- | --- | --- | --- | --- | --- | --- | --- | --- | --- | --- | --- | --- | --- | --- | --- | --- | --- | --- | --- | --- | --- | --- | --- | --- | --- | --- | --- | --- | --- | --- | --- | --- | --- | --- | --- | --- | --- | --- | --- | --- | --- | --- | --- | --- | --- | --- | --- | --- | --- | --- | --- | --- | --- | --- | --- | --- | --- | --- | --- | --- | --- | --- | --- | --- | --- | --- | --- | --- | --- | --- | --- | --- | --- | --- | --- | --- | --- | --- | --- | --- | --- | --- | --- | --- | --- | --- | --- | --- | --- | --- | --- | --- | --- | --- | --- | --- | --- | --- | --- | --- | --- | --- | --- | --- | --- | --- | --- | --- | --- | --- | --- | --- | --- | --- | --- | --- | --- | --- | --- | --- | --- | --- | --- | --- | --- | --- | --- | --- | --- | --- | --- | --- | --- | --- | --- | --- | --- | --- | --- | --- | --- | --- | --- | --- | --- | --- | --- | --- | --- | --- | --- | --- | --- | --- | --- | --- | --- | --- | --- | --- | --- | --- | --- | --- | --- | --- | --- | --- | --- | --- | --- | --- | --- | --- | --- | --- | --- | --- | --- | --- | --- | --- | --- | --- | --- | --- | --- | --- | --- | --- | --- | --- | --- | --- | --- | --- | --- | --- | --- | --- | --- | --- | --- | --- | --- | --- | --- | --- | --- | --- | --- | --- | --- | --- | --- | --- | --- | --- | --- | --- | --- | --- | --- | --- | --- | --- |
| **Gene code** | **Length** | **Domain information** || gi|32472094|ref|NP\_865088.1| | 504 | Pkinase     85-344 |
|  |  | TM     o457-479i- |
| gi|32475633|ref|NP\_868627.1| | 510 | Pkinase     77-342 |
|  |  | TM     o484-506i- |
| gi|32477250|ref|NP\_870244.1| | 1167 | Pkinase     144-403 |
| gi|32477546|ref|NP\_870540.1| | 518 | Pkinase     83-346 |
|  |  | TM     i380-402o417-439i446-468o483-505i- |
| gi|32476872|ref|NP\_869866.1| | 856 | Pkinase     118-378 |
| gi|32473866|ref|NP\_866860.1| | 543 | Pkinase     12-275 |
|  |  | TM     i342-364o- |
| gi|32476111|ref|NP\_869105.1| | 564 | Pkinase     105-363 |
|  |  | TM     o402-424i429-448o463-485i494-516o526-548i- |
| gi|32471474|ref|NP\_864467.1| | 657 | Kdo     21-201 |
|  |  | Pkinase     29-285 |
|  |  | TM     o422-444i- |
| gi|32475108|ref|NP\_868102.1| | 1044 | Pkinase     79-347 |
|  |  | RDD     357-527 |
|  |  | TM     o364-386i398-420o483-505i782-804o935-957i969-991o1006-1025i- |
| gi|32475891|ref|NP\_868885.1| | 1030 | Kdo     103-274 |
|  |  | Pkinase     105-369 |
|  |  | SBP\_bac\_10     832-1014 |
|  |  | TM     i415-437o- |
| gi|32471645|ref|NP\_864638.1| | 1073 | Pkinase     29-316 |
|  |  | WD40     455-492 |
|  |  | WD40     542-579 |
|  |  | WD40     669-706 |
|  |  | WD40     717-757 |
|  |  | WD40     976-1013 |
|  |  | TM     i345-367o- |
| gi|32473469|ref|NP\_866463.1| | 1813 | Pkinase     47-304 |
|  |  | DUF323     1537-1803 |
| gi|32475309|ref|NP\_868303.1| | 780 | Pkinase     72-338 |
|  |  | TM     i384-406o- |
| gi|32476809|ref|NP\_869803.1| | 912 | Pkinase     123-408 |
|  |  | TPR     535-568 |
|  |  | TPR     583-616 |
|  |  | TPR     634-667 |
|  |  | TPR     738-771 |
|  |  | TPR     786-819 |
|  |  | TPR     834-867 |
| gi|32471643|ref|NP\_864636.1| | 1083 | Pkinase     27-317 |
|  |  | WD40     453-490 |
|  |  | WD40     540-577 |
|  |  | WD40     669-706 |
|  |  | WD40     718-757 |
|  |  | WD40     976-1013 |
|  |  | TM     i344-366o- |
| gi|32476260|ref|NP\_869254.1| | 766 | Pkinase     154-412 |
|  |  | Kdo     167-319 |
| gi|32475439|ref|NP\_868433.1| | 513 | Pkinase     127-384 |
| gi|32476054|ref|NP\_869048.1| | 656 | Pkinase     103-384 |
| gi|32476927|ref|NP\_869921.1| | 1899 | Pkinase     384-671 |
|  |  | WD40     1118-1154 |
| gi|32476285|ref|NP\_869279.1| | 1097 | Pkinase     96-388 |
|  |  | WD40     517-553 |
|  |  | WD40     559-596 |
|  |  | WD40     601-636 |
|  |  | WD40     778-816 |
|  |  | WD40     887-923 |
|  |  | WD40     1012-1048 |
| gi|32475672|ref|NP\_868666.1| | 488 | Pkinase     103-367 |
|  |  | TM     o386-408i- |
| gi|32473790|ref|NP\_866784.1| | 1290 | Pkinase     99-397 |
|  |  | TPR     957-990 |
|  |  | TPR     991-1024 |
|  |  | TPR     1028-1061 |
| gi|32477961|ref|NP\_870955.1| | 783 | Pkinase     450-741 |
|  |  | TM     i87-109o405-427i- |
| gi|32475155|ref|NP\_868149.1| | 611 | Pkinase     143-388 |
|  |  | TM     o329-351i447-469o479-501i526-548o558-580i- |
| gi|32476268|ref|NP\_869262.1| | 364 | Pkinase     10-278 |
|  |  | TM     o338-360i- |
| gi|32474038|ref|NP\_867032.1| | 769 | Pkinase     87-336 |
|  |  | TPR     521-554 |
|  |  | TPR     662-695 |
|  |  | TM     i366-385o- |
| gi|32474490|ref|NP\_867484.1| | 966 | Pkinase     104-385 |
| gi|32476735|ref|NP\_869729.1| | 781 | Pkinase     89-386 |
|  |  | TM     i416-438o- |
| gi|32471646|ref|NP\_864639.1| | 1090 | Pkinase     49-338 |
|  |  | WD40     484-521 |
|  |  | WD40     571-608 |
|  |  | WD40     701-738 |
|  |  | WD40     749-789 |
|  |  | WD40     1007-1044 |
|  |  | TM     i368-390o- |
| gi|32477437|ref|NP\_870431.1| | 758 | Pkinase     89-387 |
| gi|32472874|ref|NP\_865868.1| | 555 | Pkinase     71-347 |
|  |  | TM     o388-410i423-445o460-479i486-508o518-540i- |
| gi|32475037|ref|NP\_868031.1| | 937 | Pkinase     113-379 |
| gi|32475300|ref|NP\_868294.1| | 364 | Pkinase     61-320 |
| gi|32474612|ref|NP\_867606.1| | 746 | Pkinase     420-685 |
|  |  | TM     i43-65o379-401i- |
| gi|32472189|ref|NP\_865183.1| | 471 | Pkinase     91-348 |
|  |  | TM     o442-464i- |
| gi|32477268|ref|NP\_870262.1| | 880 | Pkinase     585-850 |
|  |  | TM     i165-187o- |
| gi|32471012|ref|NP\_864005.1| | 779 | Pkinase     86-384 |
| gi|32477592|ref|NP\_870586.1| | 812 | Pkinase     79-354 |
|  |  | TPR     511-544 |
|  |  | TPR     728-761 |
|  |  | TM     o384-406i- |
| gi|32476375|ref|NP\_869369.1| | 417 | Response\_reg     1-123 |
|  |  | Kdo     134-333 |
|  |  | Pkinase     138-406 |
| gi|32473279|ref|NP\_866273.1| | 344 | Pkinase     23-331 |
| gi|32471862|ref|NP\_864856.1| | 797 | Pkinase     147-410 |
|  |  | PAS     536-602 |
| gi|32470744|ref|NP\_863737.1| | 987 | Pkinase     148-456 |
| gi|32476406|ref|NP\_869400.1| | 693 | Pkinase     44-296 |
|  |  | TM     o398-420i494-516o529-551i563-585o605-627i639-656o666-685i- |
| gi|32475823|ref|NP\_868817.1| | 1922 | Pkinase     285-559 |
|  |  | WD40     920-957 |
|  |  | WD40     962-998 |
|  |  | WD40     1059-1098 |
|  |  | WD40     1132-1170 |
|  |  | WD40     1184-1222 |
|  |  | WD40     1252-1289 |
|  |  | WD40     1312-1350 |
|  |  | WD40     1403-1440 |
|  |  | WD40     1641-1675 |
|  |  | WD40     1680-1719 |
|  |  | WD40     1730-1810 |
|  |  | WD40     1826-1863 |
|  |  | WD40     1879-1916 |
|  |  | TM     o476-498i673-695o- |
| gi|32472416|ref|NP\_865410.1| | 327 | Pkinase     27-319 |
| gi|32471608|ref|NP\_864601.1| | 802 | Pkinase     52-316 |
|  |  | TM     o749-771i- |
| gi|32472791|ref|NP\_865785.1| | 502 | Kdo     32-244 |
|  |  | Pkinase     72-337 |
| gi|32477775|ref|NP\_870769.1| | 305 | Pkinase     28-293 |
| gi|32471783|ref|NP\_864777.1| | 314 | Pkinase     46-288 |
| gi|32476378|ref|NP\_869372.1| | 553 | Pkinase     9-272 |
|  |  | TM     o333-352i- |
| gi|32476397|ref|NP\_869391.1| | 1097 | Pkinase     37-286 |
| gi|32470905|ref|NP\_863898.1| | 375 | Pkinase     49-338 |
| gi|32472478|ref|NP\_865472.1| | 1998 | Pkinase     17-270 |
|  |  | TPR     684-717 |
|  |  | TPR     780-813 |
|  |  | TPR     1051-1084 |
|  |  | TPR     1143-1176 |
|  |  | HisKA     1431-1498 |
|  |  | HATPase\_c     1545-1660 |
|  |  | Response\_reg     1693-1814 |
|  |  | Hpt     1894-1977 |
| gi|32477661|ref|NP\_870655.1| | 343 | Pkinase     84-320 |
| gi|32475105|ref|NP\_868099.1| | 793 | Pkinase     208-521 |
|  |  | TM     o547-569i586-608o634-656i668-690o725-744i751-773o- |
| gi|32472951|ref|NP\_865945.1| | 1287 | Kdo     49-215 |
|  |  | Pkinase     54-334 |
| gi|32474384|ref|NP\_867378.1| | 1128 | Pkinase     117-363 |
| gi|32473307|ref|NP\_866301.1| | 570 | ABC1     127-246 |
|  |  | TM     i512-534o538-560i- |
| gi|32475572|ref|NP\_868566.1| | 585 | ABC1     132-251 |
|  |  | TM     i518-540o560-582i- |
