# Supplementary material for: Diversity in domain architectures of Ser/Thr kinases and their homologues in prokaryotes
Source: BMC Genomics. 2005 Sep 19;6:129. doi: 10.1186/1471-2164-6-129 (PMC1262709; doi:10.1186/1471-2164-6-129)
Supplement: Additional File 1 — Data files comprising of the description of protein kinases and homologues encoded in genomes of organisims considered in the current analysis are provided as supplementary information accompanying this article. Each additional data file lists the gene identifiers, length, and domain arrangement of protein kinases and homologues identified in the current analysis. [file 1471-2164-6-129-S1.tar › Supplementary_files/Pseudomonas_aeruginosa.htm]

Kinases in Pseudomonas aeruginosa


# Kinases in Pseudomonas aeruginosa

|  |  |  |  |  |  |  |  |  |  |  |  |  |  |  |  |  |  |  |  |  |  |  |  |  |  |  |  |  |  |  |  |  |  |  |  |  |  |  |  |  |  |  |  |  |  |  |  |  |  |  |  |  |  |  |  |  |  |  |  |  |  |  |
| --- | --- | --- | --- | --- | --- | --- | --- | --- | --- | --- | --- | --- | --- | --- | --- | --- | --- | --- | --- | --- | --- | --- | --- | --- | --- | --- | --- | --- | --- | --- | --- | --- | --- | --- | --- | --- | --- | --- | --- | --- | --- | --- | --- | --- | --- | --- | --- | --- | --- | --- | --- | --- | --- | --- | --- | --- | --- | --- | --- | --- | --- | --- |
| **Gene code** | **Length** | **Domain information** || gi9945898gbAAG03464.1AE004446\_12 | 1032 | Pkinase     8-259 |
|  |  | VWA     615-799 |
| gi9947642gbAAG05060.1AE004594\_12 | 329 | Pkinase     51-316 |
| gi9947763gbAAG05171.1AE004604\_1 | 531 | SpoIIE     10-205 |
|  |  | Pkinase     237-487 |
|  |  | TM     o507-529i- |
| gi9948555gbAAG05890.1AE004677\_7 | 499 | LRR     90-112 |
|  |  | LRR     113-135 |
|  |  | LRR     158-177 |
|  |  | LRR     180-202 |
|  |  | LRR     203-225 |
|  |  | Pkinase     256-488 |
| gi9948803gbAAG06115.1AE004700\_11 | 1210 | Pkinase     113-355 |
| gi9951045gbAAG08166.1AE004891\_8 | 297 | RIO1     30-226 |
| gi9951357gbAAG08450.1AE004919\_11 | 533 | ABC1     114-233 |
| gi9951294gbAAG08393.1AE004913\_8 | 244 | Kdo     15-219 |
| gi9951292gbAAG08391.1AE004913\_6 | 492 | Kdo     281-469 |
| gi9951293gbAAG08392.1AE004913\_7 | 252 | Kdo     22-224 |
| gi9951283gbAAG08383.1AE004912\_9 | 216 | Kdo     7-196 |
| gi9951295gbAAG08394.1AE004913\_9 | 268 | Kdo     20-231 |
