# Supplementary material for: Diversity in domain architectures of Ser/Thr kinases and their homologues in prokaryotes
Source: BMC Genomics. 2005 Sep 19;6:129. doi: 10.1186/1471-2164-6-129 (PMC1262709; doi:10.1186/1471-2164-6-129)
Supplement: Additional File 1 — Data files comprising of the description of protein kinases and homologues encoded in genomes of organisims considered in the current analysis are provided as supplementary information accompanying this article. Each additional data file lists the gene identifiers, length, and domain arrangement of protein kinases and homologues identified in the current analysis. [file 1471-2164-6-129-S1.tar › Supplementary_files/Pseudomonas_putida KT2440.htm]

Kinases in Pseudomonas putida KT2440


# Kinases in Pseudomonas putida KT2440

|  |  |  |  |  |  |  |  |  |  |  |  |  |  |  |  |  |  |  |  |  |  |  |  |  |  |  |  |  |  |  |  |  |  |  |  |  |  |  |  |  |  |  |  |  |  |  |  |  |  |  |  |  |  |  |  |  |  |  |  |  |  |  |  |  |  |  |  |  |  |  |  |  |  |  |  |  |  |  |  |  |
| --- | --- | --- | --- | --- | --- | --- | --- | --- | --- | --- | --- | --- | --- | --- | --- | --- | --- | --- | --- | --- | --- | --- | --- | --- | --- | --- | --- | --- | --- | --- | --- | --- | --- | --- | --- | --- | --- | --- | --- | --- | --- | --- | --- | --- | --- | --- | --- | --- | --- | --- | --- | --- | --- | --- | --- | --- | --- | --- | --- | --- | --- | --- | --- | --- | --- | --- | --- | --- | --- | --- | --- | --- | --- | --- | --- | --- | --- | --- | --- | --- |
| **Gene code** | **Length** | **Domain information** || gi|24983617|gb|AAN67705.1|AE016401\_8 | 556 | PP2C     5-221 |
|  |  | Pkinase     275-508 |
|  |  | TM     o532-554i- |
| gi|26988816|ref|NP\_744241.1| | 556 | PP2C     5-221 |
|  |  | Pkinase     275-508 |
|  |  | TM     o532-554i- |
| gi|24987042|gb|AAN70807.1|AE016724\_6 | 1682 | Pkinase     24-271 |
|  |  | NACHT     326-533 |
|  |  | GAF     1280-1423 |
|  |  | HisKA     1461-1531 |
|  |  | HATPase\_c     1572-1680 |
| gi|26991918|ref|NP\_747343.1| | 1682 | Pkinase     24-271 |
|  |  | NACHT     326-533 |
|  |  | GAF     1280-1423 |
|  |  | HisKA     1461-1531 |
|  |  | HATPase\_c     1572-1680 |
| gi|24984515|gb|AAN68520.1|AE016484\_2 | 297 | RIO1     30-226 |
| gi|26989631|ref|NP\_745056.1| | 297 | RIO1     30-226 |
| gi|24986707|gb|AAN70504.1|AE016692\_3 | 224 | Kdo     34-221 |
| gi|26991615|ref|NP\_747040.1| | 224 | Kdo     34-221 |
| gi|24981713|gb|AAN65976.1|AE016226\_5 | 244 | Kdo     11-219 |
| gi|26987087|ref|NP\_742512.1| | 244 | Kdo     11-219 |
| gi|24981714|gb|AAN65977.1|AE016226\_6 | 250 | Kdo     22-223 |
| gi|26987088|ref|NP\_742513.1| | 250 | Kdo     22-223 |
| gi|24981715|gb|AAN65978.1|AE016226\_7 | 480 | Kdo     281-461 |
| gi|26987089|ref|NP\_742514.1| | 480 | Kdo     281-461 |
