# Supplementary material for: Diversity in domain architectures of Ser/Thr kinases and their homologues in prokaryotes
Source: BMC Genomics. 2005 Sep 19;6:129. doi: 10.1186/1471-2164-6-129 (PMC1262709; doi:10.1186/1471-2164-6-129)
Supplement: Additional File 1 — Data files comprising of the description of protein kinases and homologues encoded in genomes of organisims considered in the current analysis are provided as supplementary information accompanying this article. Each additional data file lists the gene identifiers, length, and domain arrangement of protein kinases and homologues identified in the current analysis. [file 1471-2164-6-129-S1.tar › Supplementary_files/Pseudomonas_syringae_pv_tomato_str_DC3000.htm]

Kinases in Pseudomonas syringae pv. tomato str. DC3000


# Kinases in Pseudomonas syringae pv. tomato str. DC3000

|  |  |  |  |  |  |  |  |  |  |  |  |  |  |  |  |  |  |  |  |  |  |  |  |  |  |  |  |  |  |  |  |  |  |  |  |  |  |  |  |  |  |  |  |  |  |  |  |  |  |  |  |  |  |  |  |  |  |  |  |  |  |  |  |  |  |  |  |  |  |  |  |  |  |  |  |  |  |  |  |  |  |  |  |  |  |  |  |  |  |  |  |  |  |  |  |  |  |  |  |  |  |  |  |  |  |  |  |  |  |  |  |  |  |  |  |  |
| --- | --- | --- | --- | --- | --- | --- | --- | --- | --- | --- | --- | --- | --- | --- | --- | --- | --- | --- | --- | --- | --- | --- | --- | --- | --- | --- | --- | --- | --- | --- | --- | --- | --- | --- | --- | --- | --- | --- | --- | --- | --- | --- | --- | --- | --- | --- | --- | --- | --- | --- | --- | --- | --- | --- | --- | --- | --- | --- | --- | --- | --- | --- | --- | --- | --- | --- | --- | --- | --- | --- | --- | --- | --- | --- | --- | --- | --- | --- | --- | --- | --- | --- | --- | --- | --- | --- | --- | --- | --- | --- | --- | --- | --- | --- | --- | --- | --- | --- | --- | --- | --- | --- | --- | --- | --- | --- | --- | --- | --- | --- | --- | --- | --- | --- | --- | --- |
| **Gene code** | **Length** | **Domain information** || gi|28852745|gb|AAO55817.1| | 529 | Pkinase     233-492 |
|  |  | TM     o506-525i- |
| gi|28869503|ref|NP\_792122.1| | 529 | Pkinase     233-492 |
|  |  | TM     o506-525i- |
| gi|28855779|gb|AAO58838.1| | 331 | Pkinase     50-315 |
| gi|28872524|ref|NP\_795143.1| | 331 | Pkinase     50-315 |
| gi|28855518|gb|AAO58578.1| | 539 | ABC1     113-232 |
|  |  | TM     o515-537i- |
| gi|28872264|ref|NP\_794883.1| | 539 | ABC1     113-232 |
|  |  | TM     o515-537i- |
| gi|28853579|gb|AAO56646.1| | 446 | LRR     26-47 |
|  |  | LRR     49-71 |
|  |  | LRR     72-94 |
|  |  | LRR     117-139 |
|  |  | LRR     140-162 |
|  |  | LRR     163-185 |
|  |  | Pkinase     214-441 |
| gi|28870332|ref|NP\_792951.1| | 446 | LRR     26-47 |
|  |  | LRR     49-71 |
|  |  | LRR     72-94 |
|  |  | LRR     117-139 |
|  |  | LRR     140-162 |
|  |  | LRR     163-185 |
|  |  | Pkinase     214-441 |
| gi|28851212|gb|AAO54290.1| | 298 | RIO1     30-226 |
| gi|28867976|ref|NP\_790595.1| | 298 | RIO1     30-226 |
| gi|28855366|gb|AAO58426.1| | 253 | Kdo     22-223 |
| gi|28872112|ref|NP\_794731.1| | 253 | Kdo     22-223 |
| gi|28855353|gb|AAO58413.1| | 226 | Kdo     21-209 |
| gi|28872099|ref|NP\_794718.1| | 226 | Kdo     21-209 |
| gi|28855367|gb|AAO58427.1| | 244 | Kdo     21-219 |
| gi|28872113|ref|NP\_794732.1| | 244 | Kdo     21-219 |
| gi|28855130|gb|AAO58191.1| | 673 | Pkinase     209-504 |
| gi|28871877|ref|NP\_794496.1| | 673 | Pkinase     209-504 |
| gi|28855365|gb|AAO58425.1| | 481 | Kdo     281-461 |
| gi|28872111|ref|NP\_794730.1| | 481 | Kdo     281-461 |
| gi|28855368|gb|AAO58428.1| | 268 | Kdo     20-231 |
| gi|28872114|ref|NP\_794733.1| | 268 | Kdo     20-231 |
