# Supplementary material for: Diversity in domain architectures of Ser/Thr kinases and their homologues in prokaryotes
Source: BMC Genomics. 2005 Sep 19;6:129. doi: 10.1186/1471-2164-6-129 (PMC1262709; doi:10.1186/1471-2164-6-129)
Supplement: Additional File 1 — Data files comprising of the description of protein kinases and homologues encoded in genomes of organisims considered in the current analysis are provided as supplementary information accompanying this article. Each additional data file lists the gene identifiers, length, and domain arrangement of protein kinases and homologues identified in the current analysis. [file 1471-2164-6-129-S1.tar › Supplementary_files/Shewanella_oneidensis_MR_1.htm]

Kinases in Shewanella oneidensis MR-1


# Kinases in Shewanella oneidensis MR-1

|  |  |  |  |  |  |  |  |  |  |  |  |  |  |  |  |  |  |  |  |  |  |  |  |  |  |  |  |  |  |  |  |  |  |  |  |  |  |  |  |  |  |  |  |  |  |  |  |  |  |  |
| --- | --- | --- | --- | --- | --- | --- | --- | --- | --- | --- | --- | --- | --- | --- | --- | --- | --- | --- | --- | --- | --- | --- | --- | --- | --- | --- | --- | --- | --- | --- | --- | --- | --- | --- | --- | --- | --- | --- | --- | --- | --- | --- | --- | --- | --- | --- | --- | --- | --- | --- |
| **Gene code** | **Length** | **Domain information** || gi|24373036|ref|NP\_717078.1| | 1376 | Pkinase     203-457 |
|  |  | Pkinase     504-755 |
| gi|24375686|ref|NP\_719729.1| | 549 | ABC1     111-231 |
|  |  | TM     i499-516o521-540i- |
| gi|24375108|ref|NP\_719151.1| | 371 | Pkinase     1-142 |
| gi|24372172|ref|NP\_716214.1| | 602 | Pkinase     44-297 |
| gi|24375503|ref|NP\_719546.1| | 447 | LRR     37-59 |
|  |  | LRR     60-82 |
|  |  | LRR     105-127 |
|  |  | LRR     128-150 |
|  |  | LRR     151-173 |
|  |  | Pkinase     207-438 |
| gi|24372091|ref|NP\_716133.1| | 653 | Pkinase     418-650 |
| gi|24375460|ref|NP\_719503.1| | 285 | RIO1     30-226 |
|  |  | Kdo     38-213 |
| gi|24376149|ref|NP\_720192.1| | 250 | Kdo     32-243 |
