# Supplementary material for: Diversity in domain architectures of Ser/Thr kinases and their homologues in prokaryotes
Source: BMC Genomics. 2005 Sep 19;6:129. doi: 10.1186/1471-2164-6-129 (PMC1262709; doi:10.1186/1471-2164-6-129)
Supplement: Additional File 1 — Data files comprising of the description of protein kinases and homologues encoded in genomes of organisims considered in the current analysis are provided as supplementary information accompanying this article. Each additional data file lists the gene identifiers, length, and domain arrangement of protein kinases and homologues identified in the current analysis. [file 1471-2164-6-129-S1.tar › Supplementary_files/Streptomyces_avermitilis_MA_4680.htm]

Kinases in Streptomyces avermitilis MA-4680


# Kinases in Streptomyces avermitilis MA-4680

|  |  |  |  |  |  |  |  |  |  |  |  |  |  |  |  |  |  |  |  |  |  |  |  |  |  |  |  |  |  |  |  |  |  |  |  |  |  |  |  |  |  |  |  |  |  |  |  |  |  |  |  |  |  |  |  |  |  |  |  |  |  |  |  |  |  |  |  |  |  |  |  |  |  |  |  |  |  |  |  |  |  |  |  |  |  |  |  |  |  |  |  |  |  |  |  |  |  |  |  |  |  |  |  |  |  |  |  |  |  |  |  |  |  |  |  |  |  |  |  |  |  |  |  |  |  |  |  |  |  |  |  |  |  |  |  |  |  |  |  |  |  |  |  |  |  |  |  |  |  |  |  |  |  |  |  |  |  |  |  |  |  |  |  |  |  |  |  |  |  |  |  |  |  |  |  |  |  |  |  |  |  |  |  |  |  |  |  |  |  |  |  |  |  |  |  |  |  |  |  |  |  |  |  |  |  |  |  |  |  |  |  |  |
| --- | --- | --- | --- | --- | --- | --- | --- | --- | --- | --- | --- | --- | --- | --- | --- | --- | --- | --- | --- | --- | --- | --- | --- | --- | --- | --- | --- | --- | --- | --- | --- | --- | --- | --- | --- | --- | --- | --- | --- | --- | --- | --- | --- | --- | --- | --- | --- | --- | --- | --- | --- | --- | --- | --- | --- | --- | --- | --- | --- | --- | --- | --- | --- | --- | --- | --- | --- | --- | --- | --- | --- | --- | --- | --- | --- | --- | --- | --- | --- | --- | --- | --- | --- | --- | --- | --- | --- | --- | --- | --- | --- | --- | --- | --- | --- | --- | --- | --- | --- | --- | --- | --- | --- | --- | --- | --- | --- | --- | --- | --- | --- | --- | --- | --- | --- | --- | --- | --- | --- | --- | --- | --- | --- | --- | --- | --- | --- | --- | --- | --- | --- | --- | --- | --- | --- | --- | --- | --- | --- | --- | --- | --- | --- | --- | --- | --- | --- | --- | --- | --- | --- | --- | --- | --- | --- | --- | --- | --- | --- | --- | --- | --- | --- | --- | --- | --- | --- | --- | --- | --- | --- | --- | --- | --- | --- | --- | --- | --- | --- | --- | --- | --- | --- | --- | --- | --- | --- | --- | --- | --- | --- | --- | --- | --- | --- | --- | --- | --- | --- | --- | --- | --- | --- | --- | --- | --- | --- | --- | --- | --- | --- | --- |
| **Gene code** | **Length** | **Domain information** || gi|29830881|ref|NP\_825515.1| | 667 | Pkinase     11-273 |
|  |  | PASTA     376-440 |
|  |  | PASTA     443-506 |
|  |  | PASTA     509-574 |
|  |  | PASTA     577-639 |
|  |  | TM     i342-364o- |
| gi|29830915|ref|NP\_825549.1| | 499 | Pkinase     19-281 |
|  |  | TM     o363-385i- |
| gi|29830914|ref|NP\_825548.1| | 550 | Pkinase     20-287 |
|  |  | PASTA     480-546 |
| gi|29832634|ref|NP\_827268.1| | 648 | Pkinase     26-281 |
|  |  | PASTA     381-446 |
|  |  | PASTA     449-514 |
|  |  | PASTA     516-583 |
|  |  | PASTA     586-648 |
|  |  | TM     i357-379o- |
| gi|29827806|ref|NP\_822440.1| | 754 | Pkinase     11-278 |
|  |  | Kdo     23-187 |
| gi|29827698|ref|NP\_822332.1| | 324 | Pkinase     26-286 |
| gi|29831548|ref|NP\_826182.1| | 726 | Pkinase     23-280 |
|  |  | TM     o472-494i- |
| gi|29833120|ref|NP\_827754.1| | 550 | Pkinase     27-281 |
|  |  | TM     o342-364i- |
| gi|29831550|ref|NP\_826184.1| | 547 | Pkinase     13-267 |
| gi|29830083|ref|NP\_824717.1| | 487 | Pkinase     1-251 |
| gi|29831643|ref|NP\_826277.1| | 573 | Pkinase     8-273 |
| gi|29828804|ref|NP\_823438.1| | 663 | Pkinase     24-270 |
| gi|29831365|ref|NP\_825999.1| | 583 | Pkinase     15-264 |
| gi|29831967|ref|NP\_826601.1| | 803 | Pkinase     18-272 |
| gi|29831260|ref|NP\_825894.1| | 732 | Pkinase     23-272 |
|  |  | PQQ     454-492 |
|  |  | PQQ     494-531 |
|  |  | PQQ     538-575 |
|  |  | PQQ     622-659 |
|  |  | PQQ     662-699 |
|  |  | TM     i346-368o- |
| gi|29831162|ref|NP\_825796.1| | 742 | Pkinase     18-282 |
| gi|29827516|ref|NP\_822150.1| | 568 | Pkinase     15-277 |
| gi|29831551|ref|NP\_826185.1| | 385 | Pkinase     19-274 |
|  |  | TM     i334-356o- |
| gi|29833662|ref|NP\_828296.1| | 692 | Pkinase     18-283 |
| gi|29833341|ref|NP\_827975.1| | 433 | Pkinase     29-277 |
|  |  | TM     i385-404o- |
| gi|29831553|ref|NP\_826187.1| | 578 | Pkinase     97-351 |
| gi|29830359|ref|NP\_824993.1| | 782 | Pkinase     16-265 |
|  |  | PQQ     429-462 |
|  |  | PQQ     464-500 |
|  |  | PQQ     502-539 |
|  |  | PQQ     546-582 |
|  |  | PQQ     590-627 |
|  |  | PQQ     636-673 |
|  |  | PQQ     677-713 |
|  |  | PQQ     716-753 |
| gi|29830869|ref|NP\_825503.1| | 564 | Pkinase     9-257 |
|  |  | TM     o542-561i- |
| gi|29830627|ref|NP\_825261.1| | 574 | Pkinase     15-259 |
| gi|29831097|ref|NP\_825731.1| | 682 | Pkinase     15-273 |
|  |  | TM     o493-515i- |
| gi|29831644|ref|NP\_826278.1| | 413 | Kdo     7-204 |
|  |  | Pkinase     9-266 |
|  |  | TM     i372-394o- |
| gi|29831918|ref|NP\_826552.1| | 834 | Pkinase     193-447 |
| gi|29830537|ref|NP\_825171.1| | 374 | Pkinase     130-360 |
| gi|29830845|ref|NP\_825479.1| | 573 | Pkinase     72-308 |
| gi|29834045|ref|NP\_828679.1| | 882 | Pkinase     235-548 |
| gi|29827180|ref|NP\_821814.1| | 178 | Pkinase     3-176 |
| gi|29831851|ref|NP\_826485.1| | 328 | Pkinase     12-274 |
| gi|29831549|ref|NP\_826183.1| | 893 | Pkinase     40-587 |
|  |  | TM     o654-676i- |
| gi|29829610|ref|NP\_824244.1| | 451 | ABC1     112-228 |
