# Supplementary material for: Diversity in domain architectures of Ser/Thr kinases and their homologues in prokaryotes
Source: BMC Genomics. 2005 Sep 19;6:129. doi: 10.1186/1471-2164-6-129 (PMC1262709; doi:10.1186/1471-2164-6-129)
Supplement: Additional File 1 — Data files comprising of the description of protein kinases and homologues encoded in genomes of organisims considered in the current analysis are provided as supplementary information accompanying this article. Each additional data file lists the gene identifiers, length, and domain arrangement of protein kinases and homologues identified in the current analysis. [file 1471-2164-6-129-S1.tar › Supplementary_files/Streptomyces_coelicolor_A3(2).htm]

Kinases in Streptomyces coelicolor A3(2)


# Kinases in Streptomyces coelicolor A3(2)

|  |  |  |  |  |  |  |  |  |  |  |  |  |  |  |  |  |  |  |  |  |  |  |  |  |  |  |  |  |  |  |  |  |  |  |  |  |  |  |  |  |  |  |  |  |  |  |  |  |  |  |  |  |  |  |  |  |  |  |  |  |  |  |  |  |  |  |  |  |  |  |  |  |  |  |  |  |  |  |  |  |  |  |  |  |  |  |  |  |  |  |  |  |  |  |  |  |  |  |  |  |  |  |  |  |  |  |  |  |  |  |  |  |  |  |  |  |  |  |  |  |  |  |  |  |  |  |  |  |  |  |  |  |  |  |  |  |  |  |  |  |  |  |  |  |  |  |
| --- | --- | --- | --- | --- | --- | --- | --- | --- | --- | --- | --- | --- | --- | --- | --- | --- | --- | --- | --- | --- | --- | --- | --- | --- | --- | --- | --- | --- | --- | --- | --- | --- | --- | --- | --- | --- | --- | --- | --- | --- | --- | --- | --- | --- | --- | --- | --- | --- | --- | --- | --- | --- | --- | --- | --- | --- | --- | --- | --- | --- | --- | --- | --- | --- | --- | --- | --- | --- | --- | --- | --- | --- | --- | --- | --- | --- | --- | --- | --- | --- | --- | --- | --- | --- | --- | --- | --- | --- | --- | --- | --- | --- | --- | --- | --- | --- | --- | --- | --- | --- | --- | --- | --- | --- | --- | --- | --- | --- | --- | --- | --- | --- | --- | --- | --- | --- | --- | --- | --- | --- | --- | --- | --- | --- | --- | --- | --- | --- | --- | --- | --- | --- | --- | --- | --- | --- | --- | --- | --- | --- | --- | --- | --- | --- | --- | --- |
| **Gene code** | **Length** | **Domain information** || gi|21222257|ref|NP\_628036.1| | 673 | Pkinase     11-273 |
|  |  | PASTA     381-445 |
|  |  | PASTA     448-511 |
|  |  | PASTA     514-580 |
|  |  | PASTA     583-649 |
|  |  | TM     i349-368o- |
| gi|21222230|ref|NP\_628009.1| | 522 | Pkinase     19-281 |
|  |  | PT     453-490 |
|  |  | TM     o365-384i- |
| gi|21225517|ref|NP\_631296.1| | 745 | Pkinase     11-278 |
|  |  | Kdo     23-187 |
| gi|21223154|ref|NP\_628933.1| | 717 | Pkinase     23-280 |
|  |  | TM     o476-498i- |
| gi|21222892|ref|NP\_628671.1| | 586 | Pkinase     15-274 |
| gi|21222231|ref|NP\_628010.1| | 556 | Pkinase     20-287 |
|  |  | PASTA     484-550 |
| gi|21220218|ref|NP\_625997.1| | 550 | Pkinase     15-269 |
| gi|21220588|ref|NP\_626367.1| | 667 | Pkinase     28-290 |
|  |  | PASTA     402-467 |
|  |  | PASTA     470-535 |
|  |  | PASTA     537-602 |
|  |  | PASTA     605-667 |
|  |  | TM     i379-398o- |
| gi|21222269|ref|NP\_628048.1| | 576 | Pkinase     9-257 |
|  |  | TM     o554-573i- |
| gi|21223156|ref|NP\_628935.1| | 599 | Pkinase     13-267 |
|  |  | TM     i381-400o- |
| gi|21221541|ref|NP\_627320.1| | 487 | Pkinase     1-251 |
| gi|21223285|ref|NP\_629064.1| | 670 | Pkinase     33-292 |
|  |  | SBP\_bac\_3     421-648 |
| gi|21222767|ref|NP\_628546.1| | 580 | Pkinase     22-270 |
|  |  | TM     i370-392o- |
| gi|21223157|ref|NP\_628936.1| | 380 | Pkinase     18-272 |
|  |  | TM     i304-326o- |
| gi|21221774|ref|NP\_627553.1| | 720 | Pkinase     22-271 |
|  |  | PQQ     394-431 |
|  |  | PQQ     439-473 |
|  |  | PQQ     474-511 |
|  |  | PQQ     610-647 |
|  |  | PQQ     650-687 |
| gi|21220714|ref|NP\_626493.1| | 686 | Pkinase     15-263 |
|  |  | WD40     349-386 |
|  |  | WD40     396-432 |
|  |  | WD40     535-571 |
|  |  | WD40     576-613 |
| gi|21222873|ref|NP\_628652.1| | 592 | Pkinase     15-264 |
| gi|21222037|ref|NP\_627816.1| | 783 | Pkinase     25-279 |
|  |  | TM     o593-615i- |
