# Supplementary material for: Diversity in domain architectures of Ser/Thr kinases and their homologues in prokaryotes
Source: BMC Genomics. 2005 Sep 19;6:129. doi: 10.1186/1471-2164-6-129 (PMC1262709; doi:10.1186/1471-2164-6-129)
Supplement: Additional File 1 — Data files comprising of the description of protein kinases and homologues encoded in genomes of organisims considered in the current analysis are provided as supplementary information accompanying this article. Each additional data file lists the gene identifiers, length, and domain arrangement of protein kinases and homologues identified in the current analysis. [file 1471-2164-6-129-S1.tar › Supplementary_files/Sulfolobus_tokodaii.htm]

Kinases in Sulfolobus tokodaii


# Kinases in Sulfolobus tokodaii

**Gene code** | **Length** | **Domain information** || gi|15922312|ref|NP\_377981.1| | 589 | TPR     4-37 |
|  |  | Pkinase     295-585 |
| gi|15921045|ref|NP\_376714.1| | 661 | TPR     234-267 |
|  |  | TPR     268-300 |
|  |  | Pkinase     363-641 |
| gi|15921012|ref|NP\_376681.1| | 701 | Pkinase     382-687 |
|  |  | TM     i12-34o44-66i73-90o105-127i134-156o171-190i195-217o- |
| gi|15920911|ref|NP\_376580.1| | 644 | Pkinase     336-643 |
|  |  | TM     i12-34o39-56i63-85o90-112i119-138o- |
| gi|15921862|ref|NP\_377531.1| | 429 | Pkinase     139-424 |
| gi|15921136|ref|NP\_376805.1| | 630 | Pkinase     326-629 |
|  |  | TM     i12-29o80-102i109-131o- |
| gi|15922772|ref|NP\_378441.1| | 514 | Pkinase     214-509 |
|  |  | TM     i9-26o30-49i56-78o- |
| gi|15920566|ref|NP\_376235.1| | 227 | Kdo     3-194 |
|  |  | Pkinase     10-207 |
|  |  | RIO1     23-203 |
| gi|15920414|ref|NP\_376083.1| | 288 | RIO1     109-281 |
| gi|15921955|ref|NP\_377624.1| | 488 | ABC1     97-215 |
|  |  | TM     o461-483i- |
| gi|15920729|ref|NP\_376398.1| | 132 | RIO1     1-119 |
