# Supplementary material for: Diversity in domain architectures of Ser/Thr kinases and their homologues in prokaryotes
Source: BMC Genomics. 2005 Sep 19;6:129. doi: 10.1186/1471-2164-6-129 (PMC1262709; doi:10.1186/1471-2164-6-129)
Supplement: Additional File 1 — Data files comprising of the description of protein kinases and homologues encoded in genomes of organisims considered in the current analysis are provided as supplementary information accompanying this article. Each additional data file lists the gene identifiers, length, and domain arrangement of protein kinases and homologues identified in the current analysis. [file 1471-2164-6-129-S1.tar › Supplementary_files/Synechocystis.htm]

Kinases in Synechocystis


# Kinases in Synechocystis

|  |  |  |  |  |  |  |  |  |  |  |  |  |  |  |  |  |  |  |  |  |  |  |  |  |  |  |  |  |  |  |  |  |  |  |  |  |  |  |  |  |  |  |  |  |  |  |  |  |  |  |  |  |  |  |  |  |
| --- | --- | --- | --- | --- | --- | --- | --- | --- | --- | --- | --- | --- | --- | --- | --- | --- | --- | --- | --- | --- | --- | --- | --- | --- | --- | --- | --- | --- | --- | --- | --- | --- | --- | --- | --- | --- | --- | --- | --- | --- | --- | --- | --- | --- | --- | --- | --- | --- | --- | --- | --- | --- | --- | --- | --- | --- |
| **Gene code** | **Length** | **Domain information** || gi1006577gnlPIDd1011377 | 505 | Pkinase     9-267 |
|  |  | TM     o326-348i- |
| gi1652588gnlPIDd1018242 | 495 | Pkinase     46-314 |
|  |  | TM     o372-394i399-416o463-485i- |
| gi1653478gnlPIDd1019124 | 574 | Pkinase     34-296 |
|  |  | Pentapeptide     454-493 |
|  |  | Pentapeptide     504-543 |
| gi1653640gnlPIDd1019285 | 631 | Pkinase     29-279 |
| gi1653955gnlPIDd1019598 | 535 | Pkinase     12-273 |
|  |  | TM     o344-366i- |
| gi1652635gnlPIDd1018288 | 614 | Pkinase     15-238 |
| gi1652753gnlPIDd1018405 | 585 | ABC1     142-261 |
|  |  | TM     i530-549o553-575i- |
| gi1652223gnlPIDd1017880 | 566 | ABC1     127-246 |
| gi1001579gnlPIDd1010857 | 681 | ABC1     161-279 |
| gi1652697gnlPIDd1018350 | 408 | ABC1     100-223 |
| gi1208478gnlPIDd1011297 | 567 | ABC1     115-238 |
|  |  | TM     o27-46i503-525o530-552i- |
