# Supplementary material for: Diversity in domain architectures of Ser/Thr kinases and their homologues in prokaryotes
Source: BMC Genomics. 2005 Sep 19;6:129. doi: 10.1186/1471-2164-6-129 (PMC1262709; doi:10.1186/1471-2164-6-129)
Supplement: Additional File 1 — Data files comprising of the description of protein kinases and homologues encoded in genomes of organisims considered in the current analysis are provided as supplementary information accompanying this article. Each additional data file lists the gene identifiers, length, and domain arrangement of protein kinases and homologues identified in the current analysis. [file 1471-2164-6-129-S1.tar › Supplementary_files/Tropheryma_whipplei_str_Twist.htm]

Kinases in Tropheryma whipplei str. Twist


# Kinases in Tropheryma whipplei str. Twist

|  |  |  |  |  |  |  |  |  |  |  |  |  |  |  |  |  |  |  |  |  |  |  |  |  |  |  |  |  |  |  |  |  |  |  |  |  |  |  |  |  |  |  |  |  |
| --- | --- | --- | --- | --- | --- | --- | --- | --- | --- | --- | --- | --- | --- | --- | --- | --- | --- | --- | --- | --- | --- | --- | --- | --- | --- | --- | --- | --- | --- | --- | --- | --- | --- | --- | --- | --- | --- | --- | --- | --- | --- | --- | --- | --- |
| **Gene code** | **Length** | **Domain information** || gi|28493744|ref|NP\_787905.1| | 585 | Pkinase     13-275 |
|  |  | TM     o345-367i- |
| gi|28493183|ref|NP\_787344.1| | 601 | Pkinase     16-271 |
|  |  | PASTA     335-398 |
|  |  | PASTA     401-462 |
|  |  | PASTA     471-535 |
|  |  | PASTA     538-601 |
|  |  | TM     i309-331o- |
| gi|28493745|ref|NP\_787906.1| | 550 | Pkinase     14-267 |
|  |  | PASTA     345-409 |
|  |  | PASTA     410-468 |
|  |  | TM     o306-328i- |
| gi|28493564|ref|NP\_787725.1| | 398 | Pkinase     27-316 |
|  |  | TM     o305-327i- |
