# Supplementary material for: Diversity in domain architectures of Ser/Thr kinases and their homologues in prokaryotes
Source: BMC Genomics. 2005 Sep 19;6:129. doi: 10.1186/1471-2164-6-129 (PMC1262709; doi:10.1186/1471-2164-6-129)
Supplement: Additional File 1 — Data files comprising of the description of protein kinases and homologues encoded in genomes of organisims considered in the current analysis are provided as supplementary information accompanying this article. Each additional data file lists the gene identifiers, length, and domain arrangement of protein kinases and homologues identified in the current analysis. [file 1471-2164-6-129-S1.tar › Supplementary_files/Vibrio_parahaemolyticus_RIMD_2210633.htm]

Kinases in Vibrio parahaemolyticus RIMD 2210633


# Kinases in Vibrio parahaemolyticus RIMD 2210633

|  |  |  |  |  |  |  |  |  |  |  |  |  |  |  |  |  |  |  |  |  |  |  |  |  |  |  |  |  |  |  |  |  |  |  |  |  |  |  |  |  |  |  |  |  |
| --- | --- | --- | --- | --- | --- | --- | --- | --- | --- | --- | --- | --- | --- | --- | --- | --- | --- | --- | --- | --- | --- | --- | --- | --- | --- | --- | --- | --- | --- | --- | --- | --- | --- | --- | --- | --- | --- | --- | --- | --- | --- | --- | --- | --- |
| **Gene code** | **Length** | **Domain information** || gi|28900899|ref|NP\_800554.1| | 714 | Pkinase     91-362 |
|  |  | TM     i361-383o- |
| gi|28896831|ref|NP\_796436.1| | 450 | Kdo     66-259 |
|  |  | Pkinase     79-323 |
|  |  | TM     o336-358i- |
| gi|28898174|ref|NP\_797779.1| | 686 | Pkinase     73-310 |
| gi|28896871|ref|NP\_796476.1| | 544 | ABC1     111-231 |
| gi|28898759|ref|NP\_798364.1| | 414 | LRR     39-61 |
|  |  | LRR     62-84 |
|  |  | LRR     107-129 |
|  |  | LRR     130-152 |
|  |  | LRR     153-175 |
|  |  | Pkinase     205-410 |
| gi|28896970|ref|NP\_796575.1| | 238 | Kdo     29-232 |
